# Pore Geometry–Driven Capture of Trace Aromatic Volatile Organic Compounds in Al-Based MOFs

**DOI:** 10.1021/acsnano.6c05710

**Published:** 2026-07-02

**Authors:** Anastasia Blokhina, Yutao Li, Iurii Dovgaliuk, Debanjan Chakraborty, Aysu Ozturk, Nency P. Domingues, Xiaoqi Zhang, Fatmah Mish Ebrahim, Bettina Baumgartner, Christian Serre, Georges Mouchaham, Berend Smit

**Affiliations:** † Laboratory of molecular simulation (LSMO), Institut des Sciences et Ingénierie Chimiques, 27218 École Polytechnique Fédérale de Lausanne (EPFL), Rue de l’Industrie 17, Sion CH-1951, Switzerland; ‡ Institut des Matériaux Poreux de Paris, École Normale Supérieure, ESPCI Paris, 26909 CNRS, PSL University, Paris 75005, France; § Van’t Hoff Institute for Molecular Sciences, 1234 University of Amsterdam, Amsterdam 1098 XH, Netherlands

**Keywords:** metal organic frameworks, pore geometry, volatile organic compound, trace aromatic VOC capture, high-throughput screening

## Abstract

Aromatic volatile organic compounds (VOCs) are toxic air pollutants that pose serious health risks even at trace concentrations. Their nonpolar character makes the design of efficient sorbents particularly challenging, as adsorption is governed mainly by weak dispersion forces. Here, we identify pore geometry as an effective structural descriptor for discovering metal–organic frameworks (MOFs) capable of efficient trace-level VOC capture. Screening a diverse set of MOFs revealed that one-dimensional channels with rhombic or square cross sections enhance host–guest interactions and promote strong affinity for aromatic molecules. Guided by this principle and sustainability-by-design criteria, we identify MIP-211­(Al) (Materials from the Institute of Porous Materials of Paris) as a top performer with among the highest toluene uptake of 4.7 mmol g^–1^ at 0.0008 *P*/*P*_0_ (31 ppm). This material combines excellent cycling stability, facile regeneration under vacuum, and scalable green synthesis. Synchrotron powder X-ray diffraction (SPXRD) and density functional theory (DFT) calculations confirm that rhombic pore geometry governs strong toluene affinity at trace amounts. Attenuated Total Reflectance Fourier Transform Infrared (ATR-FTIR) spectroscopy showed that MIP-211­(Al) retains 60% of its performance up to 15% relative humidity. Applying pore geometry as a descriptor in a secondary screening identified a candidate with a toluene uptake of 3.6 mmol g^–1^ at 0.0008 *P*/*P*_0_ (31 ppm) at dry conditions, with potential applicability up to 70% relative humidity. This study demonstrates the importance of pore geometry as a nanoscale parameter for efficient trace aromatic VOC adsorption.

## Graphical abstract

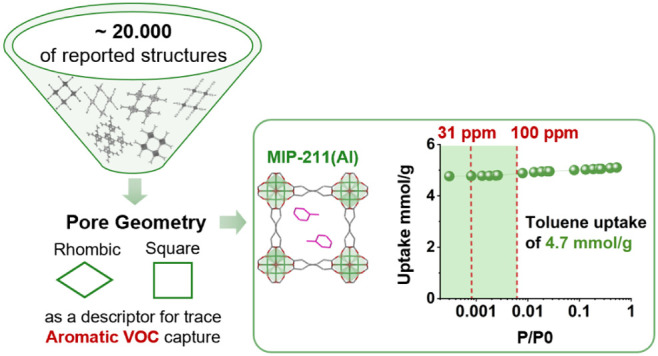

Benzene, toluene, ethylbenzene, and xylene are important aromatic hydrocarbons extensively used in the chemical industry.^1–4^ Classified as volatile organic compounds (VOCs) with hazardous properties, these molecules are often released into the atmosphere, leading to substantial air pollution and adverse health effects.^5–8^ Consequently, environmental and regulatory agencies worldwide have established ppm-level limits for indoor and outdoor VOC concentrations to mitigate their hazardous impacts.^9–11^ However, removing VOCs at such low concentrations remains a challenge due to inherently weak host–guest interactions. To address this challenge, metal–organic frameworks (MOFs) have emerged as particularly promising materials due to their structural versatility, high porosity, efficient regeneration, and scalable synthesis.^12–19^

MOF’s sorption performance depends on structural parameters such as pore volume (*V*_*p*_) and surface area (*S*_*A*_).^20^ As a result, several MOFs with high *V*_*p*_ and *S*_*A*_ have been reported^21–23^ to outperform the aromatic VOCs uptake (at *P*/*P*_0_ = 0.5) of common commercial sorbents (see Supporting Information Figure S1, Table S1). However, in the low-pressure regime (*P*/*P*_0_ = 0.005), the correlation between VOC uptake and *S*_*A*_/*V*_*p*_ breaks down, as adsorption becomes governed primarily by host–guest interactions within the pore environment.^24,25^ To improve MOF’s performance, previous studies have focused on strengthening host–guest interactions by introducing structural defects, incorporating single-atom metal sites, or functionalizing the framework with chloro groups.^16,26–28^ Despite promising results, the synthesis conditions of these materials raise sustainability concerns for potential industrial applications.^18^ To address these limitations, many studies have focused on discovering promising materials using high-throughput computational screening. This approach has been widely applied in gas adsorption studies, including VOC capture.^29–32^ For example, Liu et al.^33^ and Zhang et al.^34^ combined materials screening with machine learning to predict toluene sorption capacities in MOFs. However, these studies mainly focused on correlations between saturation uptake and common descriptors such as *S*_*A*_, *V*_*p*_, and open metal sites (OMS), while the low-pressure regime was overlooked. Yuan et al.^35^ investigated benzene adsorption at low relative pressure (*P*/*P*_0_ = 0.008) and identified promising candidates with predicted capacities surpassing reported MOFs and commercial sorbents.

These computational studies, however, often overlook practical limitations. They report hypothetical or synthetically challenging structures, highlighting the need for experimental validation. In addition, to the best of our knowledge, no structural descriptor has yet been established for accurately predicting MOF performance in the low-pressure regime. Therefore, the rational design of efficient, sustainable materials for trace-level removal of aromatic VOCs remains a significant challenge.

In this study, by combining computational screening of MOFs based on Henry coefficients of toluene/benzene with systematic literature analysis, we established pore geometry as an effective structural descriptor governing enhanced uptake at trace concentrations. Specifically, one-dimensional channels with rhombic or square geometries represent an optimal pore architecture. Guided by these insights and sustainability-by-design principles, six Al-based MOFs were selected for experimental validation of their predicted sorption performance, with toluene used as a representative model for aromatic VOC removal due to benzene’s high carcinogenicity. MIP-211­(Al)—an aluminum muconate framework [Al­(OH)­(muc)],^36^ was found to have the highest toluene uptake of 4.7 mmol g^–1^ at 31 ppm (0.0008 *P*/*P*_0_) according to static sorption measurement. Synchrotron powder X-ray diffraction (SPXRD) and density functional theory (DFT) calculations were used to identify the toluene binding sites and validate the pore-geometry-induced enhancement in sorption performance. Attenuated total reflectance Fourier-transform infrared (ATR-FTIR) spectroscopy was employed to assess the influence of water on sorption behavior under humid conditions. Furthermore, a secondary screening that incorporated pore geometry revealed a promising hydrophobic MOF candidate with uptake of 3.7 mmol g^–1^. Overall, our integrated workflow, combining high-throughput screening and experimental validation using pore geometry as a new descriptor, can be adapted for trace removal of other aromatic VOCs.

## Results and Discussion

### Screening of Structural Trends in MOFs

To investigate the potential structural properties associated with enhanced low-pressure sorption, we conducted a preliminary high-throughput screening based on the Henry coefficient (*K*_H_) of aromatic VOC as a commonly used descriptor.^35,37^ To ensure that the screening reflects only the intrinsic affinity of the frameworks toward aromatic VOCs, interactions with N_2_, O_2_, and H_2_O were not considered. We screened CoRE MOF database,^38,39^ a commonly used database containing 20,000 experimentally reported MOF structures and ranked them by simulated Henry coefficients for toluene and benzene^40^ (see Supporting Information). We did not extend our work to databases of hypothetical MOFs,^41^ which may contain materials that outperform the ones we have screened. Notably, the ten top-ranked materials share a common pore geometry: one-dimensional channels with rhombic or square cross-sections Figure 1. Similar pore architectures have been reported in microporous MOFs exhibiting efficient aromatic VOC capture.^15,37,42–44^ In addition, channel dimensionality, geometric confinement, and pore chemistry have been shown to enhance host–guest interactions between VOC molecules and porous materials, including MOFs and zeolites.^45–50^ However, while these effects have been discussed in the context of adsorption and separation performance, the specific relationship between pore geometry and trace-level aromatic VOC adsorption in MOFs has not yet been systematically investigated.

**Figure 1:**
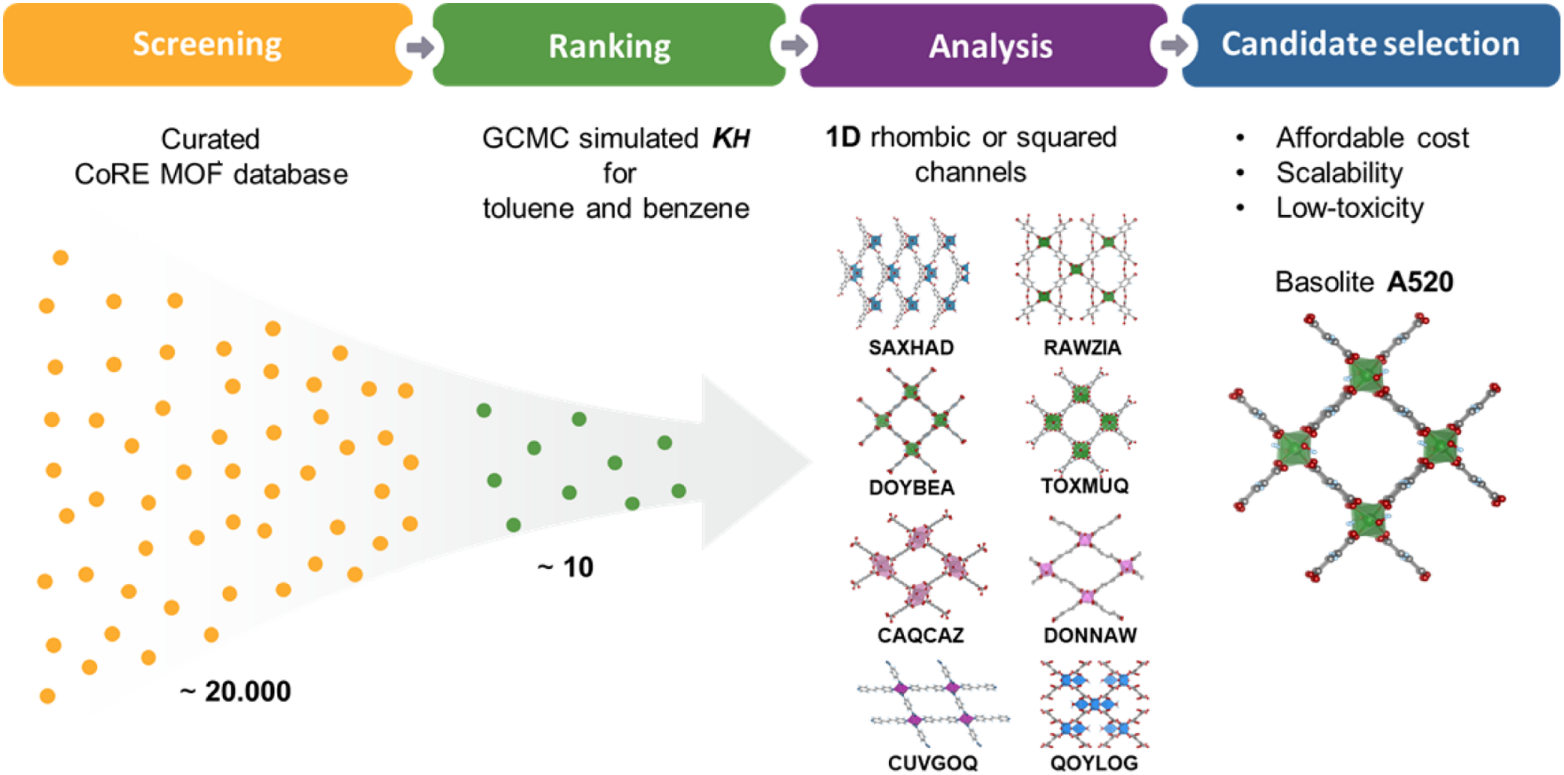
Systematic selection of materials for efficient aromatic VOCs capture at low concentrations, integrating computational screening methods with fundamental chemical principles.

To select candidates for experimental validation, we further manually assessed these structures based on metal node abundance, synthesis conditions, and structural stability Table S2. The following structures—SAHXAD, DOYBEA, RAWZIA, TOXMUQ, and OPUDIN—contain metal nodes based on low-toxicity and cost-effective Al or Zn and are synthesized using H_2_O as the primary solvent. Despite their potential for large-scale production, many of these materials may face structural stability challenges during pore activation. Considering these factors, DOYBEA, identified as MIL-53­(Al)-fumarate (commercially available as Basolite A520), was selected as the most promising candidate from this initial screening, owing to its sustainable and scalable synthesis, high performance, and crystal structure stability.^51^

### Manual Expansion of the Candidate MOF Space

Based on computational screening and literature analysis, we propose pore geometry as a novel performance metric for capturing aromatic VOCs at trace concentrations. To validate this hypothesis, we expanded our search beyond the top-performer to include other reported structures with rhombic or square channels. For environmental relevance, materials were selected following sustainability-by-design principles,^52–54^ prioritizing abundant, low-toxicity Al-MOFs with scalable and environmentally friendly synthesis routes. Furthermore, other well-known descriptors in MOFs adsorption include the pore limiting diameter (*PLD*), which ensures pore accessibility, and *S*_*A*_, which governs sorption capacity.^55–59^ Given that the kinetic diameters of benzene, toluene, ethylbenzene, and *p*-xylene range from 5.9 to 6 Å,^60^ the *PLD* in this case should exceed this value to prevent diffusion limitations. Therefore, beyond the inclusion of pore geometry, we opted for Al-MOFs showing increasing *S*_*A*_ with a minimum pore diameter of 6 Å, bearing in mind the possible flexibility of MOF structures. To achieve this, we selected linkers of systematically increasing length that incorporate π–bonds, which are essential for promoting strong host–guest interactions.^61,62^ The selected linkers are *t,t*-muconate, terephthalate, fumarate, isophthalate, and 2,5-furandicarboxylate.

We identified two structural groups. The first group includes MIL-53­(Al)-muconate,^63^ MIL-53­(Al)^64^ and MIL-53­(Al)-fumarate,^51^ which share an orthorhombic crystal system. In these materials, trans-μ–OH-bridged, corner-sharing AlO_6_ octahedra form rhomb-shaped one-dimensional channels.^64^ The second group consists of tetragonal structures with a *cis* configuration, such as MIP-211­(Al),^36^ CAU-10­(Al),^65^ and MIL-160­(Al)^66^ Figure 2, which result in square-shaped channels. Additionally, scalable and environmentally friendly synthesis routes have been reported for all the aforementioned MOFs, with the exception of MIL-53­(Al)-muconate.^36,51,67–69^ Based on the *S*_*A*_ values in Table S3, the saturation capacities of the selected MOF candidates are expected to follow the order: MIP-211­(Al) > MIL-53­(Al) > MIL-53­(Al)-muconate > MIL-53­(Al)-fumarate > CAU-10­(Al) > MIL-160­(Al). At trace concentrations, the ranking should remain the same, assuming that the pore geometry results in only a minor capacity drop between the high- and low-pressure regimes, with the exception of MIL-53­(Al) due to its structural flexibility.^64^

**Figure 2:**
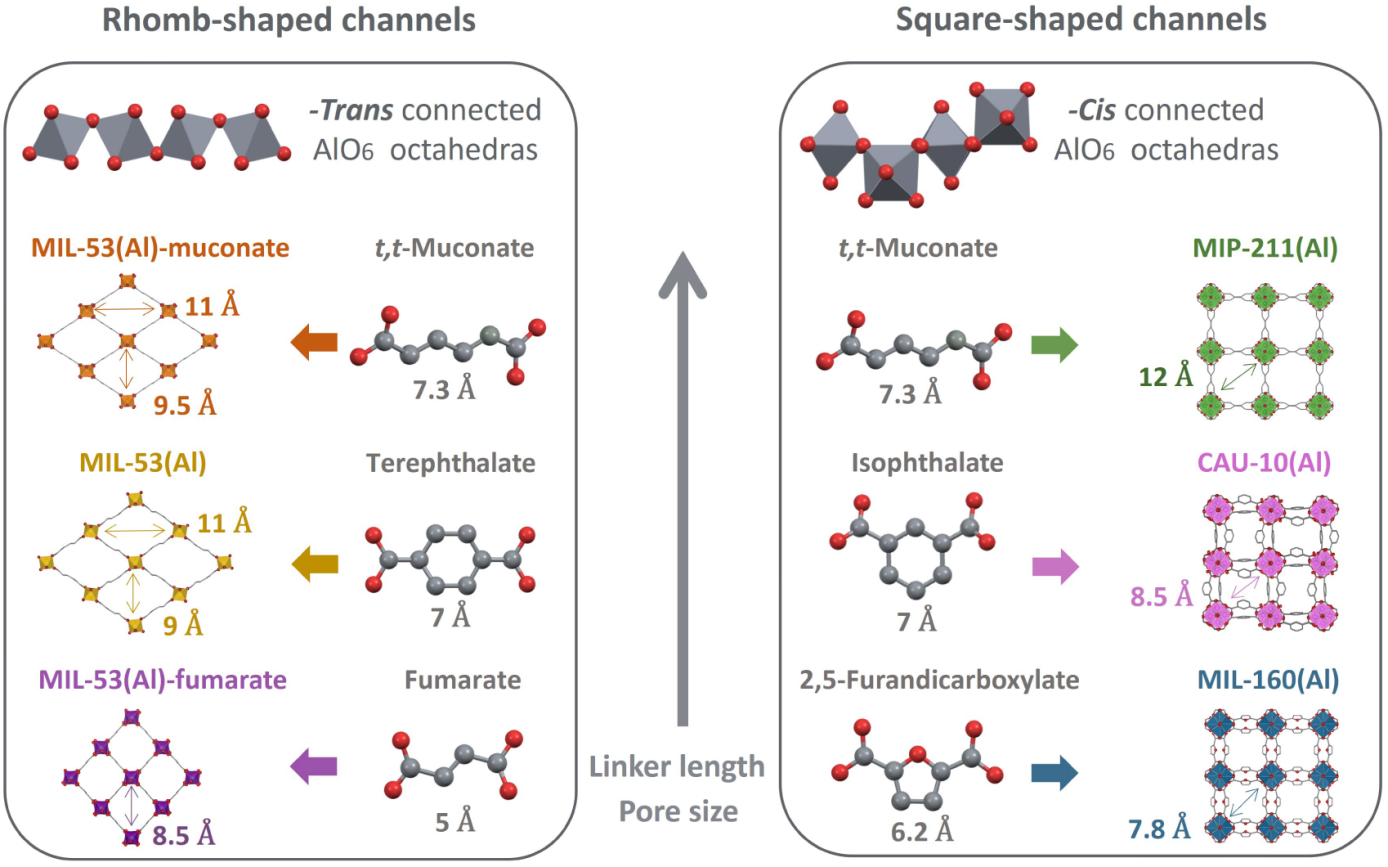
Chemical Expansion of Al-MOF candidates using various linker molecules. MOFs are categorized into two groups based on their channel type. Within each group, the linker length and corresponding MOF pore size increase progressively from bottom to top. Framework atoms (ball-and-sticks) are colored as follows: C-gray, O-red. The AlO_6_ (octahedras) are shown in different colors depending on the MOF: MIL-53­(Al)-muconate-orange, MIL-53­(Al)-yellow, MIL-53­(Al)-fumarate-purple, MIP-211­(Al)-green, CAU-10­(Al)-pink, MIL-160­(Al)-blue.

### Experimental Validation of Computational Predictions and Chemical Design Principles

Selected MOF candidates were synthesized according to previously reported protocols.^36,51,66,68,70^ The phase purity of the obtained materials was confirmed *via* powder X-ray diffraction (PXRD) Figure S2, where the experimental PXRD patterns are in agreement with the theoretical ones. Chemical degradation temperature was assessed with thermal gravimetric analysis (TGA) Figure S3. Surface area and pore volume were calculated for N_2_ sorption isotherms using Brunauer–Emmett–Teller (BET) analysis Figure S4, Figure S5, Table S3. For experimental validation, we chose toluene^71^ as a representative aromatic VOC and a safer alternative to carcinogenic benzene.^72^ The toluene uptake of the MOF candidates was measured using a gravimetric sorption method. With the current sorption technique, we assess the uptake of toluene ranging from 0.0003 (12 ppm, low-pressure) to 0.55 (21150 ppm, high pressure) of *P*/*P*_0_ Figure 3. As discussed earlier, the saturation uptake is consistent with the expectations based on *S*_*A*_: MIP-211­(Al) (5.1 mmol g^–1^), MIL-53­(Al) (4.6 mmol g^–1^), MIL-53­(Al)-muconate (3.6 mmol g^–1^), MIL-53­(Al)-fumarate (3 mmol g^–1^), CAU-10­(Al) (2.5 mmol g^–1^) and MIL-160­(Al) (2.2 mmol g^–1^) Figure 3a. The same trend is observed at lower pressures (below *P*/*P*_0_ = 0.01), the uptake ranking remains the same, except for MIL-53­(Al), where the uptake drops from 4.6 mmol g^–1^ to 1 mmol g^–1^ Figure 3b, due to the flexibility of the crystal structure. Similar breathing effect of MIL-53­(Al) was also observed during Xe and H_2_ adsorption.^73,74^

**Figure 3:**
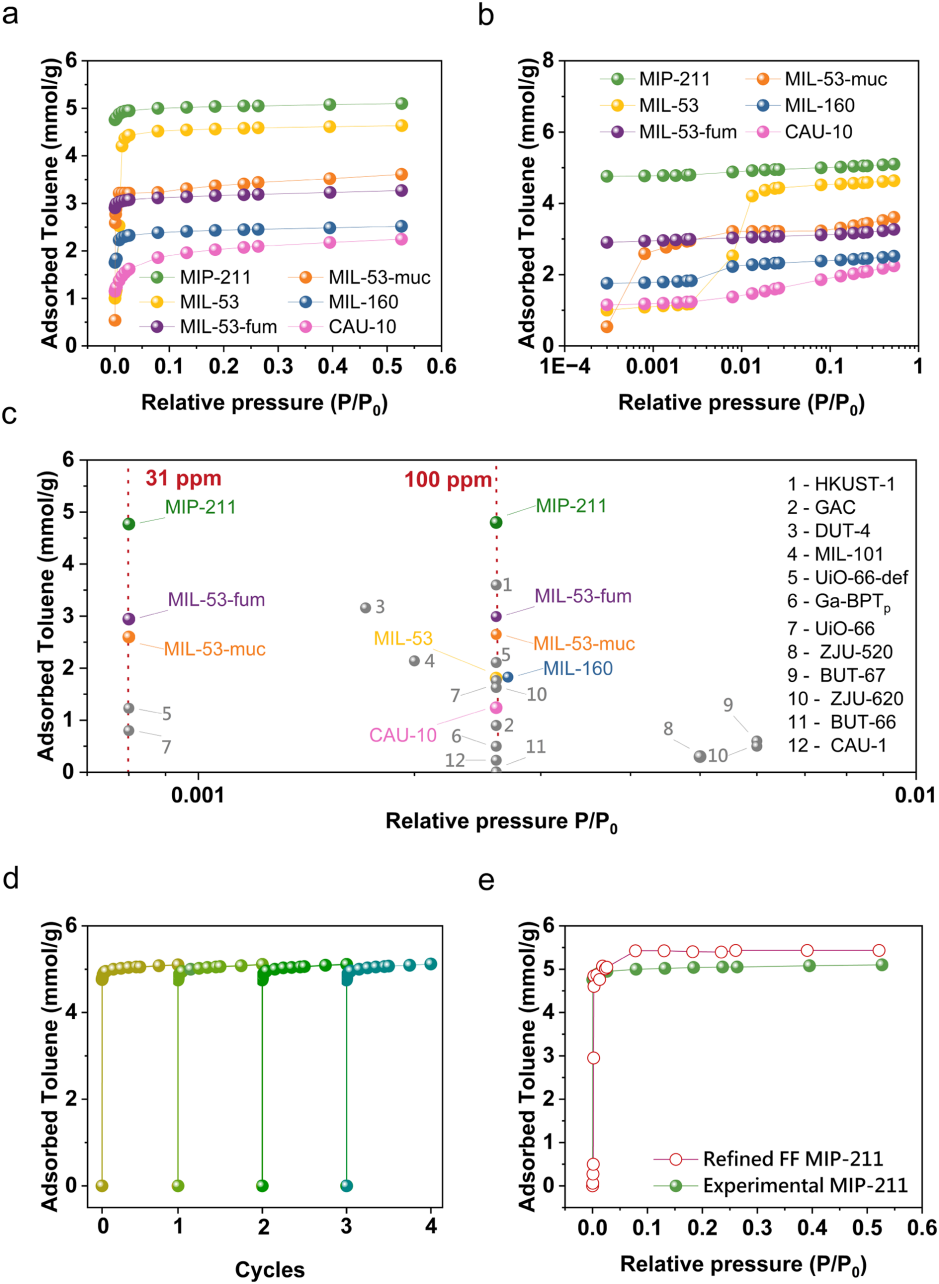
Testing of candidates. a) Toluene capture isotherms for the preselected candidates at linear scale, b) Toluene capture isotherms at logarithmic scale, c) Toluene uptake comparison with the literature data (SI) based on static sorption data (GAC-granulated activated carbon), d) Cyclic experiment for MIP-211­(Al) e) Simulated toluene isotherm using refined universal force field (UFF) versus experimental data for MIP-211­(Al).

The desorption curves for all tested MOFs were collected using the gravimetric sorption method under secondary vacuum and are presented in Figure S6. Figure 3c compares the low-pressure toluene uptake of the reported MOFs, where MIP-211­(Al) displays the highest uptake, reaching 4.8 mmol g^–1^ at 100 ppm and outperforming all previously reported structures under static sorption conditions. In addition, at *P*/*P*_0_ = 0.0008 (31 ppm) of toluene, the sorption capacity of MIP-211­(Al) is 4 times higher compared to the benchmark activated carbon. Comparisons for other pressures *P*/*P*_0_ = 0.01 and 0.1 are shown on Table S4.

To investigate the regenerability of MIP-211­(Al), we performed cyclic toluene adsorption–desorption experiments. No thermal treatment was applied during the desorption process, and regeneration was achieved solely under secondary vacuum for 1 h Figure 3d. During four cycles, MIP-211­(Al) showed no change in uptake within the entire pressure range. In addition, the retention of the crystal structure and porosity after cycling confirms the material’s stability Figure S7. Even under atmospheric pressure, flushing with N_2_ restored 87% of the MOF’s capacity Figure S8. In contrast, the regeneration of commonly used activated carbon remains challenging, as it requires elevated temperatures and suffers from performance degradation with each cycle.^75^ Structural stability during toluene desorption under the vacuum up to 500 K was investigated using high-resolution SPXRD (Figure S13, Figure S16). Among all candidates, MIP-211­(Al) emerges as the most efficient material for trace toluene removal, combining the highest uptake at low pressure with facile low-energy regeneration.

To assess the predictive capability of our approach, we further simulated the toluene isotherms using a refined universal force field (UFF) model previously developed by our group.^76^ For MIL-53­(Al)-fumarate Figure S10a,b the simulated isotherms show excellent agreement with the experimental data. In case of MIP-211­(Al) Figure 3e, Figure S10f the simulated uptake underestimates the experimental values only at pressures below *P*/*P*_0_ = 0.002. At such low pressures, the simulation contains only a small number of toluene molecules within the framework. In contrast, the predicted uptake for MIL-53­(Al)-muconate Figure S10c,d significantly exceeds the experimental value. We assume that this MOF might have a deviation from its reported crystal structure due to rotation effects of the linker (t,t-muconic acid) as we observe an additional reflection below 2θ = 10° on the XRD pattern Figure S2b, that has not been characterized in the original publication.^63^ MIL-53­(Al) Figure S11a,b and MIL-160­(Al) Figure S11c,d, show two-step experimental isotherms, indicating the structure flexibility,^73,77^ which results in over- or under-prediction in different pressure regimes. In case of CAU-10­(Al) Figure S11e,f, the simulated isotherm matches well the experimental one in the low-pressure regime, but underpredicts at higher pressures because of the rotational flexibility of the aromatic linker rings.^78^ Therefore, the obtained results indicate that our model reliably describes toluene adsorption in MOFs. Nevertheless, for low-pressure applications, computational models should incorporate potential structural flexibility to ensure accurate predictions.

### Binding Sites of Toluene

So far, we have experimentally confirmed that the pore geometry affects the affinity of the MOF for toluene. However, we now aim to identify the specific MOF–toluene interactions responsible for this high sorption performance. These insights will help establish criteria for selecting promising materials in the future.

To elucidate the preferential binding sites of toluene in MIP-211­(Al), we conducted high-resolution synchrotron powder X-ray diffraction (SPXRD) on a toluene-saturated sample, complemented by density functional theory (DFT) calculations. The toluene positions were determined from SPXRD *via* direct space method in FOX^79^ Figure S14 and refined using the Rietveld method in Fullprof,^80^ resulting in one symmetrically independent molecule with the crystallographic site occupancy of $\frac{1}{2}$ Figure S14. The arrangement of the toluene can be described pairs of molecules which facing each other *via* methyl groups Figure 4a. The $\frac{1}{2}$ of crystallographic occupancy indicates the 50% probability to observe each of such molecules and corresponds to 1 toluene per Al in the crystal structure. Overlaying the DFT-optimized toluene positions (gold) with experimental crystal structure (pink) in Figure 4b shows that toluene molecules primarily occupy the corners of the pores, near both the metal nodes and the organic linkers, and are evenly distributed within these regions. The proposed DFT model does not indicate the formation of π-stacked toluene molecules Figure S15, suggesting that the above-mentioned arrangement of the pairs is more favorable.

**Figure 4:**
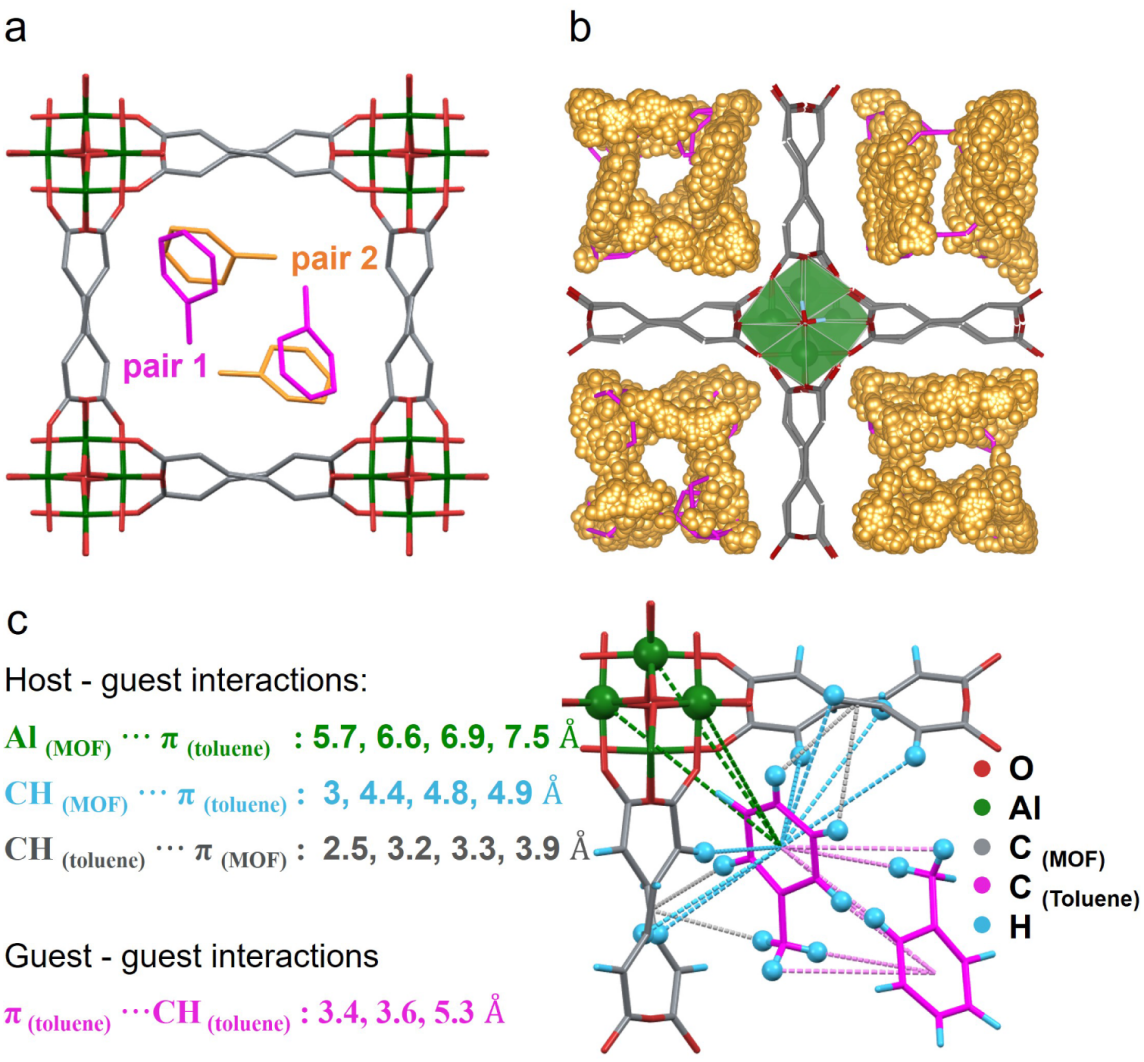
Toluene binding sites a) possible toluene positions in the pores of MIP-211­(Al) according to SPXRD, b) DFT calculated positions of toluene carbons (golden) overlapped with experimental toluene carbons (pink), c) host–guest and guest–guest interactions in MIP-211­(Al) loaded with toluene. Atoms involved in the interactions are shown as spheres.

To rationalize the preferential adsorption sites, we analyzed the nature of host–guest interactions within MIP-211 (Al). As shown in Figure 4c, the Al^3+^ centers in the metal nodes engage in cation···π interactions with the aromatic rings of toluene, at distances of approximately 5.66, 6.59, 6.89, and 7.48 Å, consistent with their high charge density.^81^ DFT calculations reveal the presence of 16 toluene molecules per unit cell. Thus, considering that the MIP-211­(Al) unit cell contains 16 Al atoms, this corresponds to an approximate 1:1 toluene-to-Al stoichiometry, which is in agreement with the experimental crystal structure model. Furthermore, additional stabilizing interactions exist between the C–H group of the linker (muconate) and the π···electron cloud of toluene’s aromatic ring at distances of 3.04, 4.38, 4.8, 4.84 Å.^82^ Similar interactions are observed between the conjugated diene system of the muconate and the C–H bonds of toluene, at 2.50, 3.22, 3.33, and 3.88 Å. Furthermore, Figure 4c illustrates toluene–toluene interactions, which are predominantly governed by C–H···π interactions between toluene molecules, with typical distances of 3.36, 3.62, 5.3 Å. These types of noncovalent interactions between MOF and aromatic VOCs were previously reported for similar systems.^24,27,42^ Therefore, square or rhombic pore geometries enhance low-pressure uptake by promoting overlapping host–guest interactions through angular confinement. This allows toluene to interact simultaneously with multiple pore walls, increasing the enthalpy of adsorption in the Henry regime, thereby strengthening physisorption under dilute conditions.

### Toluene Uptake under Humid Conditions

Until now, all performance metrics were considered under secondary vacuum and at 0% humidity. However, to demonstrate the applicability of our top candidate MIP-211­(Al) as a toluene sorbent, its performance under humid conditions needs to be evaluated as well. Although breakthrough experiments and binary mixture adsorption isotherms can assess performance under humid conditions, they provide primarily macroscopic information and do not reveal molecular-level interactions.^83–85^ For this reason, we employed *in situ* ATR-FTIR spectroscopy, which enables direct observation of toluene sorption under controlled humidity and varying flow rates Figure 5.^86,87^ In the current study, a constant flow of N_2_ gas mixed with toluene or water vapor is circulated through a closed cell where the MOF is deposited onto the surface of the ATR crystal (details of the setup and sample preparation can be found in SI). Sorption of toluene is then assessed by evaluating the C=C stretch vibration of the aromatic ring of toluene in the recorded FTIR spectra (1495 cm^–1^) Figure 5a, Figure S18d,f. Water sorption, instead, is evaluated by monitoring its O–H stretching (2700 to 3700 cm^–1^) in combination with data from water isotherm Figure S18c,e, Figure S17. Figure 5a shows differential spectra of toluene as a function of the partial pressure. To confirm the feasibility of quantitative analysis using this technique, we first compared the isotherm generated *via* ATR-FTIR spectroscopy with that obtained from static sorption analysis. The two data sets show excellent agreement within the experimental error, validating the reliability of the spectroscopic approach Figure 5c.

**Figure 5:**
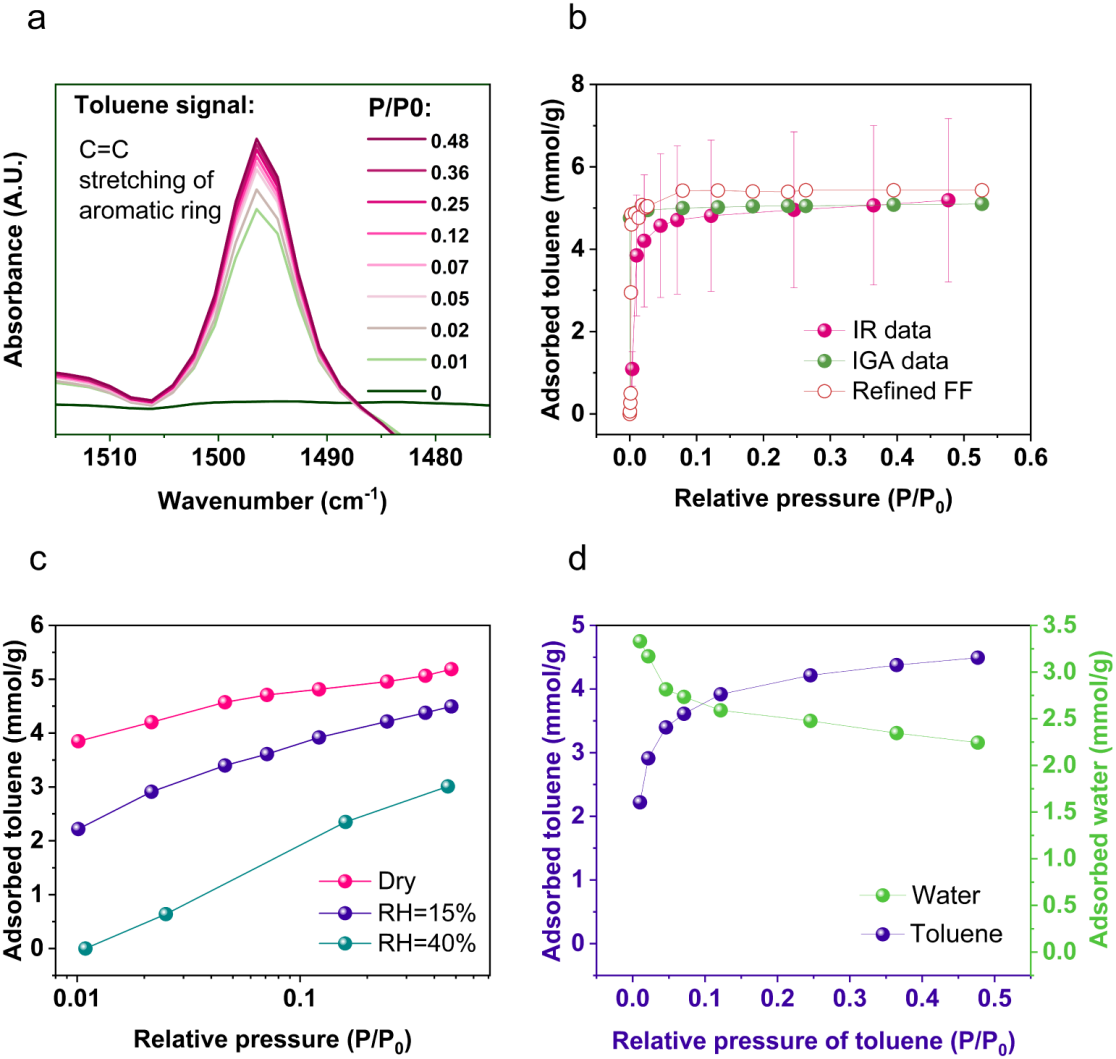
a) FTIR spectra of toluene in MIP-211­(Al) at different partial pressures of toluene, b) Toluene adsorption isotherms for MIP-211­(Al): calculated (red), static sorption (green), ATR-FTIR (pink), c) ATR-FTIR toluene isotherms under dry conditions (pink), RH = 15% (blue), RH = 40% (cyan), d) ATR-FTIR toluene isotherm (blue) at constant RH = 15% and changes in water uptake (green) at constant RH = 15% depending on partial pressure of toluene.

With our ATR-FTIR technique validated, we turned to our relative humidity (RH) experiments. To show how strongly water influences the performance of the MOF, we exposed the material at RH from 0 to 40% before introducing toluene vapors Figure 5c. Based on the water isotherm data for MIP-211­(Al) Figure S17, we picked 15% (minimal water uptake) of RH and 40% (maximum water uptake) for further analysis. As a result, at *P*/*P*_0_ = 0.01 in case of RH = 40% the MOF loses its sorption capacity toward toluene, while at RH = 15% half of the initial uptake can be recovered. Finally, by quantifying the water uptake at RH = 15% (presuming the highest water signal is the water uptake of MOF at RH = 15%, obtained from water isotherm Figure S17), we show that toluene supplants 33% of water molecules at *P*/*P*_0_ = 0.5 Figure 5d. In addition, the differential spectra Figure S18 show a gradual increase in the Al–OH band at 3683 cm^–1^, indicating displacement of water molecules from μ–OH sites by toluene, consistent with its higher binding energy (toluene: −56.65 kJ mol^–1^, water: −45 kJ mol^–1^).^36^ Quantification for RH = 40% is presented on the Figure S18b. Thus, for potential applications, MIP-211­(Al) can be employed as a sorbent for the trace removal of VOCs, in combination with industrial dehumidification methods that reduce the relative humidity to approximately up to 15%, such as molecular-sieve (zeolites) drying^88^ or cooling and condensation systems,^89,90^ which often utilize existing plant water-cooling infrastructure.

### Secondary MOF Screening: Pore Geometry as a Descriptor

Since pore geometry was identified as a key descriptor for enhanced aromatic VOC capture at trace concentrations, we conducted a secondary screening of MOFs to validate our descriptor and identify additional candidates. The MOF database was refined to include only structures with one-dimensional channels and angular confinement (see Supporting Information). Then, we applied the MOFchecker package, previously reported by our group,^91^ to automatically remove charged structures and retain only MOFs containing Al, In, or Ga nodes (some In- and Ga-based structures may have Al analogues). To evaluate toluene adsorption performance, for both the initial and secondary screenings, Grand Canonical Monte Carlo (GCMC) simulations were performed to simulate the *K*_H_ for toluene. Additionally, in the secondary screening, GCMC simulations of toluene uptake at P/P_0_ = 0.0026 (Figure S12a) were carried out. Previously studied materials such as MIP-211­(Al), MIL-53­(Al)-fumarate, MIL-53­(Al) MIL-160 and CAU-10 were also included. However, *K*_*H*(*toluene*)_ showed poor correlation with loading. For example, CAU-10 exhibits a higher *K*_*H*(*toluene*)_ (61 mol kg^–1^Pa^–1^) than MIP-211 (0.09 mol (kgPa)^−1^), despite near-saturation low-pressure uptake in both cases. This discrepancy arises because the *K*_*H*_ calculations consider only a single toluene molecule within the MOF and therefore do not capture adsorption behavior at high loadings. Since many MOFs exhibit high sorption capacities at *P*/*P*_0_ = 0.0026, the simulated toluene loading at this pressure was selected as the primary descriptor for ranking the candidates. This approach identified XAJQAC (In-TBAPy) (Figure S12b) with one-dimensional rhombic channels as a top candidate. Although its Sr analogue has been reported for benzene capture under humid conditions, performance at trace concentrations has not yet been explored.^92^ Therefore, we measured toluene adsorption of its Al analogue (Al-TBAPy), which shows an uptake of 4 mmol g^–1^ at P/P_0_ = 0.0026 (100 ppm) (Figure S12c,d, Figure S9). The material also exhibits high hydrophobicity up to 70%.^57^ Thus, Al-TBAPy emerges as a potential alternative to MIP-211­(Al) for aromatic VOC removal under humid conditions.

## Conclusions

This work identifies pore geometry as a key nanoscale descriptor governing the adsorption of aromatic VOCs at trace concentrations. High-throughput screening and structural analysis reveal that top-performing MOFs share one-dimensional channels with rhombic or square cross-sections, which enhance host–guest interactions in the physisorption regime. Guided by this insight, MIP-211­(Al) was identified as a highly efficient adsorbent, exhibiting toluene uptakes of 4.8 mmol g^–1^ at 100 ppm and 4.7 mmol g^–1^ at 31 ppm. Under dry conditions, it outperforms activated carbon while maintaining good regenerability and stability under low humidity. Its performance arises from multiple Al-π and CH-π interactions enabled by angular pore confinement. Extending this descriptor to a secondary screening identified Al-TBAPy, a hydrophobic MOF potentially suitable for humid conditions up to RH = 70%. More broadly, this work highlights pore geometry as a powerful design parameter for discovering MOFs capable of capturing aromatic VOCs at trace concentrations.

## Methods

### Materials Synthesis

MIL-53­(Al)-fumarate was synthesized following the method previously described by Álvarez et al.^51^ A mixture of Al_2_(SO_4_)_3_·18H_2_O (0.77 mmol, 513 mg), fumaric acid (1.54 mmol, 179 mg), urea (1.54 mmol, 92 mg) and deionized water (278 mmol, 5 mL) was transferred into a 23 mL Teflon-lined steel Parr autoclave. The reaction was carried out at 110 °C for 32 h, with a controlled heating ramp of 0.15 °C min^–1^. The resulting white solid was collected, washed three times with ethanol, and dried in air at room temperature.

MIL-53­(Al)-muconate was synthesized following the method previously reported.^36^ A mixture of Al­(NO_3_)_3_·9H_2_O (3.2 mmol, 1.2 g), t,t-muconic acid (3.52 mmol, 0.5 g), deionized water (11.2 mL), and dimethylformamide (3.8 mL) was transferred into a 23 mL Teflon-lined steel Parr autoclave. The reaction was carried out at 120 °C for 6 h. The resulting solid product was collected by centrifugation, washed three times with DMF and water, and dried in air at room temperature.

MIP-211­(Al) was synthesized following the method previously reported.^36^ A mixture of Al_2_(SO_4_)_3_·18H_2_O (3.26 mmol, 2.176 g), t,t-muconic acid (3.26 mmol, 0.464 g), deionized water (15 mL), and dimethylformamide (5 mL) was transferred into a 100 mL round-bottom flask. The reaction was stirred under reflux for 6 h. The resulting solid product was collected by centrifugation, washed three times with DMF and water, and dried in air at room temperature.

MIL-53­(Al) was synthesized following the method previously reported.^70^ A mixture of AlCl_3_·6H_2_O (150 mmol, 36.15 g) and H_2_O (200 mL) was transferred into a 500 mL round-bottom flask and heated at 100 °C. Then terephtalic acid (125 mmol, 20.75 g) was dissolved in 2 M NaOH (120 mL) and added to the flask under continuous stirring. The reaction was carried out under reflux for 12 h. The resulting solid product was collected by filtration and dried in air at room temperature overnight. The free linker from the sample was removed by calcination under air at 330 °C.

CAU-10­(Al) was synthesized following the method previously reported.^68^ A mixture of m-Na_2_BDC-solution (0.5 M, 2.52 mol) and ethanol (420 mL) was transferred into a round-bottom flask (10 L) equipped with a heating mantle. Then Al_2_(SO_4_)_3_ (1.89 L, 0.5 M, 0.945 mol) and NaAlO_2_ (1.26 L, 0.5 M, 0.63 mol) were added to the flask under stirring. The reaction was carried out under the reflux for 10 h. The resulting solid product was collected by hot filtration and washed with H_2_O (30 L). Then the obtained solid was redispersed in H_2_O (5 L), heated under reflux for 1 h, collected by hot filtration, and dried at 100 °C for 12 h.

MIL-160­(Al) was synthesized following the method previously reported.^66^ A mixture of Al­(OH)­(CH_3_COO)_2_ (75 mmol, 11.71 g), 2,5-furandicarboxylic acid (75 mmol, 12.16 g), and H_2_O (75 mL) was transferred into a round-bottom flask (250 mL). The reaction was carried out under reflux for 24 h. The resulting solid product was collected by filtration, washed three times with ethanol, and dried in the oven at 100 °C.

Al-TBAPy was synthesized following the method previously reported.^57^ Al­(NO_3_)_3_·9H_2_O (0.03 mmol, 11.3 mg), 1,3,6,8-tetrakis­(p-benzoicacid)­pyrene (TBAPy) (0.02 mmol, 10 mg), DMF/dioxane/H_2_O (4 mL, ratio 2/1/1) and HCl (32 wt%, 10 μL) was transferred into glass vial (12 mL) and sonicated. The reaction was carried out at 85 °C for 12 h. The resulting solid product was collected by filtration, washed three times with DMF and acetone, and dried in air at room temperature.

### Convenient Powder X-ray Diffraction

PXRD data for all samples were obtained using a Bruker D8 Advance diffractometer at room temperature with monochromated Cu Kα radiation (λ = 1.5418 Å). The measurements were conducted with a 2θ step size of 0.02° across varying θ ranges. Simulated PXRD patterns were generated from the respective crystal structures using Mercury 3.0, while structural images were created with VESTA (4.6.0).

### Synchrotron Powder X-ray Diffraction

SPXRD data were obtained at BM-01 from the Swiss-Norwegian beamlines (SNBL) located at the European Synchrotron Radiation Facility (ESRF) in Grenoble (France). The multifunctional diffractometer, based on the Dectris Pilatus 2X detector,^93^ combined with a gas-dosing system (for dynamic vacuum) and a Cryostream 700+ heater, has been used. The samples were filled up to 1 mm capillaries and connected to the gas-loading system, heated from 300 to 500 K under dynamic vacuum with further cooling back to 300 K (6 K/min rate). The diffractograms were recorded every 20 s.

### Thermogravimetric Analysis

The decomposition temperature of the samples was determined using a PerkinElmer Thermogravimetry Analyzer. The analysis was carried out under a gas flow consisting of 80% air and 20% N_2_, with a heating rate of 5 °C/min up to a maximum temperature of 700 °C.

### Nitrogen Sorption

Nitrogen adsorption isotherm measurements at 77 K were conducted using a BELSORP Mini (BEL Japan, Inc.). Prior to measurement, the samples were activated under a dynamic vacuum: MIL-53­(Al)-fumarate, MIL-53­(Al), CAU-10­(Al) and MIL-160­(Al) at 150 °C for 12 h, MIL-53­(Al)-muconate and MIL-211­(Al) at 175 °C for 7 h, and Al-TBAPy at 170 °C for 12 h.

### Toluene Sorption

Toluene adsorption measurements were recorded using single component gravimetric vapor sorption analyzer IGA-002 (Hiden Isochema) at 25 °C. Before the measurement the samples were thermally pretreated under ultra high vacuum (10^–8^ mbar): MIL-53­(Al)-fumarate, MIL-53­(Al), CAU-10­(Al) and MIL-160­(Al) at 150 °C for 12 h, MIL-53­(Al)-muconate and MIL-211­(Al) at 175 °C for 7 h, Al-TBAPy at 170 °C for 12 h.

### Water Sorption

The volumetric water sorption measurements were performed using a Micromeritics-triflex instrument. Before the measurements, the sample was degassed using a Micromeritics SmartVacPrep degas unit: heating up to 180 °C on the degas port (secondary vacuum: P = 10^–6^ mbar), at which point the outgassing rate was ≤2 μbar min^–1^. The air in the solvent reservoir was removed by freezing the solvent with liquid nitrogen and applying a vacuum at the same time. The solvent was then allowed to melt, and the process was continued twice to remove all the dissolved gases in the solvent. The temperature during the isotherm measurements was maintained using a chiller unit from Micromeritics

### FTIR-ATR Spectroscopy

Toluene adsorption under humid conditions was monitored using an Invenio-R FTIR spectrometer (Bruker Optics, Ettlingen, Germany) equipped with a custom-made gas flow cell and N_2_-cooled MCT detector in the sample compartment. To perform adsorption experiments under controlled humidity conditions, the gas flow cell was connected *via* separate tubing to water and toluene reservoirs. A N_2_ supply was distributed through mass flow controllers (MFCs), which regulated the gas streams through the water and toluene reservoirs. This setup enabled precise control of both the relative humidity and the partial pressure of toluene introduced into the gas flow cell. Similar setup was reported previously.^86,87^ For sample preparation, 10 mg of the MOF was dispersed in 2 mL of anhydrous hexane, and 100 μL of the resulting suspension was drop-cast onto the ATR crystal surface. All manipulations were performed under an inert atmosphere inside a glovebox to prevent the MOF from being exposed to humidity. Prior to each measurement, the MOF on the ATR crystal was activated under vacuum at 175 °C for 7 h using a Schlenk line. The activated sample was then returned to the glovebox for assembly in the FTIR-ATR flow cell, which was subsequently transferred outside the glovebox and connected to the sample compartment of the FTIR spectrometer for the measurement. The acquired spectra were analyzed with the OPUS 7.5 software package (Bruker Optics), while further spectral processing and fitting were carried out using MATLAB.

## Supplementary Material



## Notes

Supplementary text of XRD patterns, TGA curves, nitrogen adsorption–desorption isotherms, surface area, pore volume and diameter, toluene desorption isotherms of MIL-53­(Al), MIL-53­(Al)-fumarate, MIL-53­(Al)-muconate, MIP-211­(Al), MIL-160­(Al), and CAU-10­(Al); XRD pattern and nitrogen adsorption–desorption isotherm of MIP-211­(Al) after four toluene adsorption–desorption cycles; water adsorption isotherm of MIP-211­(Al); SPXRD pattern and Rietveld refinement plot of toluene-loaded MIP-211­(Al); XRD pattern and nitrogen adsorption–desorption isotherm of Al-TBAPy; quantification in ATR-FTIR spectroscopy; ATR-FTIR spectra of MIP-211­(Al) loaded with toluene at RH = 15% and 40%; Henry coefficient calculation, GCMC-generated toluene isotherms of MIL-53­(Al), MIL-53­(Al)-fumarate, MIL-53­(Al)-muconate, MIP-211­(Al), MIL-160­(Al), and CAU-10­(Al); MOFs screening based on Henry coefficient and toluene loading using refined database (PDF)
