# Supplementary material for: Pore Geometry–Driven Capture of Trace Aromatic Volatile Organic Compounds in Al-Based MOFs
Source: ACS Nano. 2026 Jul 2;20(27):19548–59. doi: 10.1021/acsnano.6c05710 (PMC13374495; doi:10.1021/acsnano.6c05710)
Supplement: Supplementary file 1 [file nn6c05710_si_001.pdf]

Supplementary Information

Pore Geometry–Driven Capture of Trace Aromatic Volatile Organic Compounds in Al-Based MOFs

Anastasia Blokhina[1], Yutao Li[1], Iurii Dovgaliuk[2], Debanjan Chakraborty[2], Aysu Ozturk[2], Nency Patricio Domingues[1], Xiaoqi Zhang[1], Fatmah Mish Ebrahim[1], Bettina Baumgartner[3], Christian Serre[2], Georges Mouchaham[2], and Berend Smit[*1]

[1]Laboratory of molecular simulation (LSMO), Institut des Sciences et Ingénierie Chimiques, École Polytechnique Fédérale de Lausanne (EPFL), Rue de l'Industrie 17, Sion CH-1951, Switzerland

[2]Institut des Matériaux Poreux de Paris, ENS, ESPCI Paris, CNRS, PSL University, Paris 75005, France

[3]Van 't Hoff Institute for Molecular Sciences, University of Amsterdam, Amsterdam 1098 XH, Netherlands

[*]*Email: berend.smit@epfl.ch*

Contents

S1 Supplementary Figures and Tables **3**

S2 Molecular simulation details **19**

S2.1 MOF Database 19

S2.2 Henry coefficient calculation 19

S2.3 Isotherm generation 19

S2.4 Database refinement based on pore geometry 23

S3 Synchrotron powder X-ray diffraction (SPXRD) experiment **24**

S4 ATR-FTIR spectroscopy **28**

S4.1 Quantification in ATR–FTIR spectroscopy 28

S4.2 Determination of the MOF amount present on the ATR crystal 29

S4.3 Determination of the toluene amount absorbed by MOF 30

S4.4 Determination of the water amount absorbed by MOF 30

S1 Supplementary Figures and Tables

The following abbreviations are commonly used to denote families of metal–organic frameworks (MOFs):

- **MIP** – Materials from Institute of Porous Materials of Paris
- **HKUST** – Hong Kong University of Science and Technology
- **MIL** – Materials of Institut Lavoisier
- **BPT** – 1,1′-biphenyl-3,4′,5-tricarboxylate
- **MFM** – Manchester Framework Material
- **UiO** – University of Oslo
- **BUT** – Beijing University of Technology
- **STA** – St Andrews Porous Material
- **DUT** – Dresden University of Technology
- **ZIF** – Zeolitic Imidazolate Framework
- **CAU** – Christian-Albrechts-Universität
- **ZJU** – Zhejiang University
- **TBAPy** – 1,3,6,8-tetrakis(p-benzoicacid)pyrene

SI Table 1: Reported benzene and toluene uptakes of selected MOFs and benchmark materials measured *via* static sorption. Capacity loss is calculated between saturation conditions $P/P_0 = 0.5$ and low-pressure conditions ($P/P_0 = 0.005$). AC-activated carbon.

	Benzene (mmol g^{-1})		Toluene (mmol g^{-1})		Capacity loss (%)		Ref.
Material	0.5	0.005	0.5	0.005	Benz.	Tol.	
MIL-101(Cr)	16.7	0.5	9.8	3.8	97	61	[1,2]
MOF-177(Zn)	15.6	0.1	–	–	99	–	[3]
BUT-12(Zr)	14	–	–	–	100	–	[4]
ZJU-520(Al)	10	1	6	0.27	90	96	[5]
HKUST-1(Cu)	9.5	1	6	4.36	90	27	[6,7]
Ga-BPT$_p$	7.5	2.8	8	6.3	63	21	[8]
MIL-125(Ti)	5.5	0.05	–	–	99	–	[9]
STA-26(Zr)	4.8	3.2	–	–	33	–	[4]
ZJU-620(Al)	4.3	2.3	6.6	0.3	47	96	[5]
MFM-300(Al)	4.3	2.3	–	–	47	–	[10]
MFM-68(Zr)	3.8	2.11	–	–	45	–	[11]
BUT-55(Co)	3	2.9	–	–	3.3	–	[12]
UiO-66(Zr)	4.3	2.3	3.27	2.04	47	38	[10,13]
DUT-4(Al)	–	–	5.3	3.8	–	28	[14]
CAU-1(Al)	–	–	5.2	0.67	–	87	[15]
Granulated AC	4.5	1.5	3.5	0.9	67	74	[16]
Zeolite 13X / NaX	2.2	1.7	2.8	1.4	23	50	[17–19]
Silica-gel	1	0.1	3.5	0.9	90	74	[17,20]

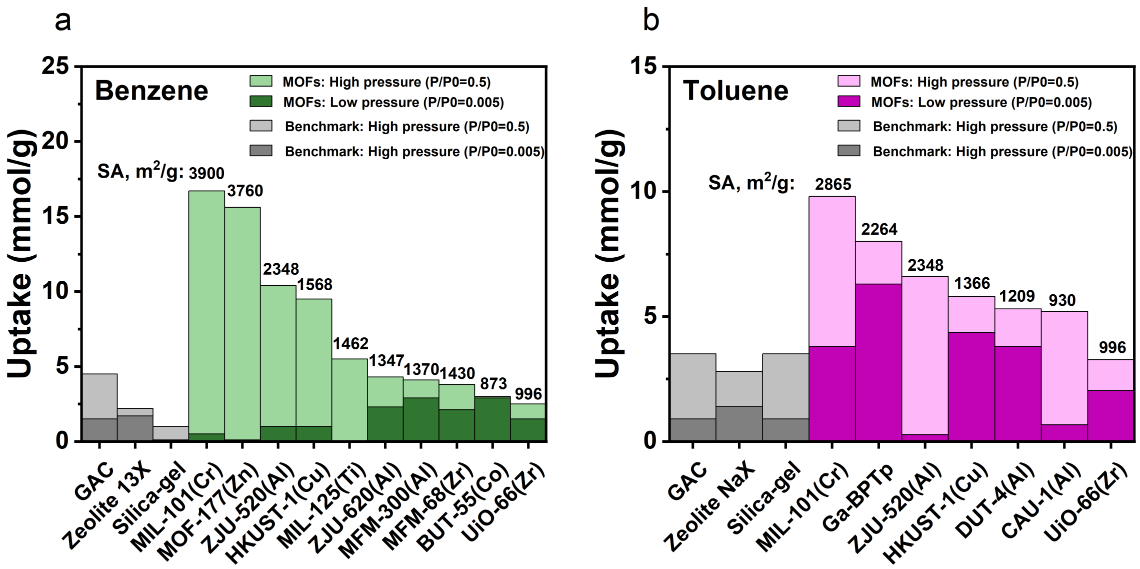

SI Fig. 1: Benzene and toluene uptake comparison for reported MOFs at different pressure regimes a) benzene at $P/P_0 = 0.5$, b) toluene at $P/P_0 = 0.5$, c) benzene at $P/P_0 = 0.005$, d) toluene at $P/P_0 = 0.005$. CSD - Cambridge Structural Database, GAC-Granulated activated carbon.

SI Table 2: Ranking of materials by Henry's coefficients for benzene and toluene, and structural data.

**CSD name**	$K_{H(benzene)}$	$K_{H(toluene)}$	**Metal**	**Solvent**	**Challenges**	**Pore geometry**
SAHXAD[21]	214617	130929	Zn	H_2O	blocked pores	rhombic channels
DOYBEA[22]	21204	21583	Al	H_2O	–	rhombic channels
IRAJER[23]	4136	4106	Ga	H_2O	charged	squared channels
RAWZIA[24]	933	934	Al	H_2O	blocked pores	rhombic channels
CAQCAZ[25]	858	843	In	DMF, H_2O	charged	rhombic channels
CUVGOQ[26]	534	533	Mn	DMF, MeOH	unstable	parallelogram channels
TOXMUQ[27]	260	260	Al	H_2O	small pores	squared channels
QOYLOG[28]	222	230	Ni	MeOH	charged	rhombic channels
OPUDIN[29]	210	205	Zn	H_2O	charged	ellipsoid channels
DONNAW[30]	183	183	Ga	H_2O	flexible structure	rhombic channels

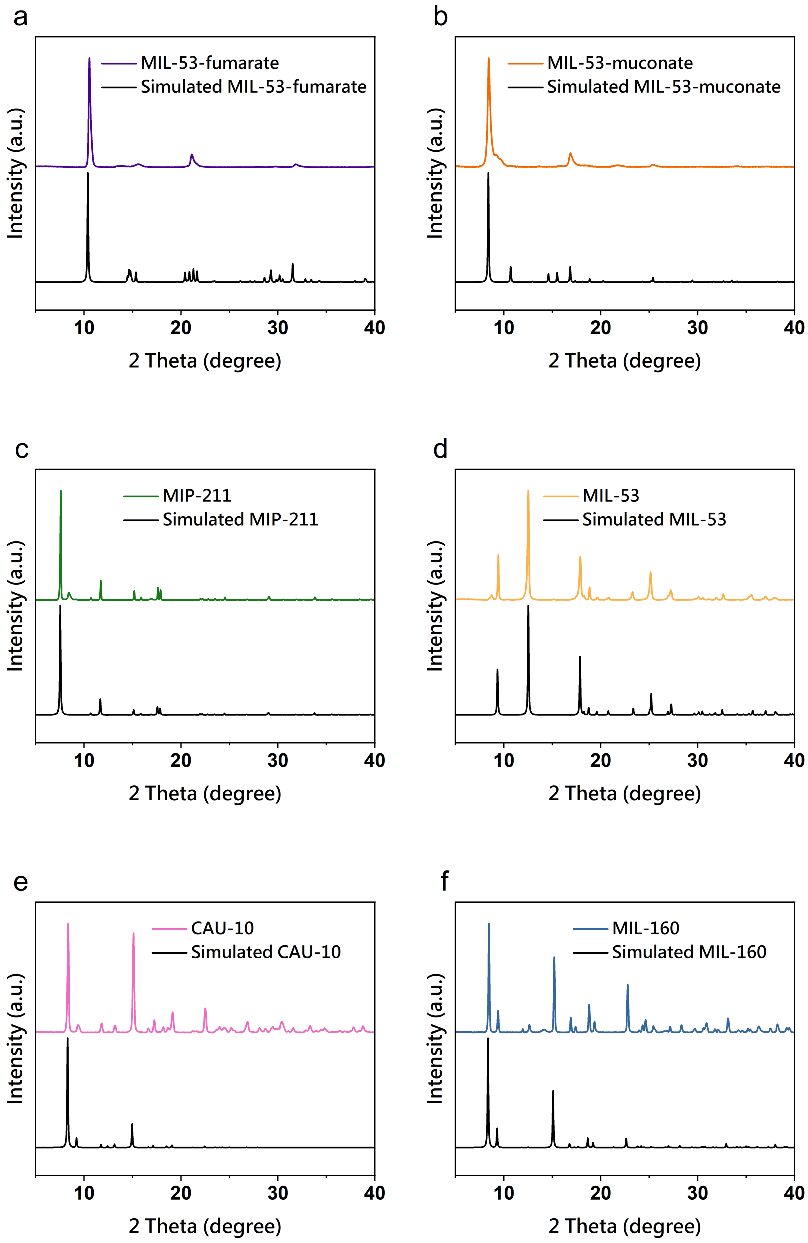

SI Fig. 2: Experimental powder x-ray diffraction patterns using Cu Kα radiation (λ = 1.5418 Å) in comparison with simulated for a) MIL-53(Al)-fumarate, b) MIL-53(Al)-muconate, c) MIP-211(Al), d) MIL-53(Al), e) CAU-10(Al), f) MIL-160(Al).

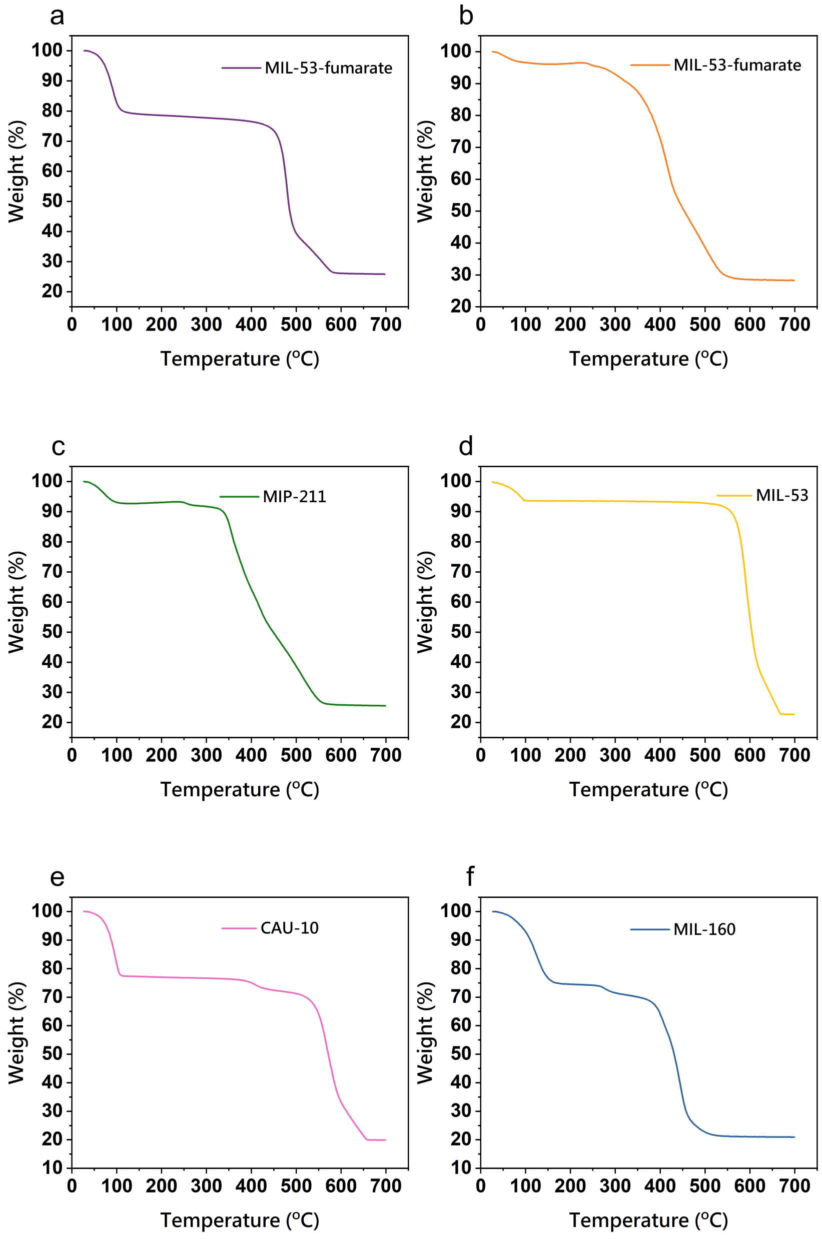

SI Fig. 3: TGA curves for a) MIL-53(Al)-muconate, b) MIL-53(Al), c) MIL-53(Al)-fumarate, d) MIP-211(Al), e) CAU-10(Al), f) MIL-160(Al).

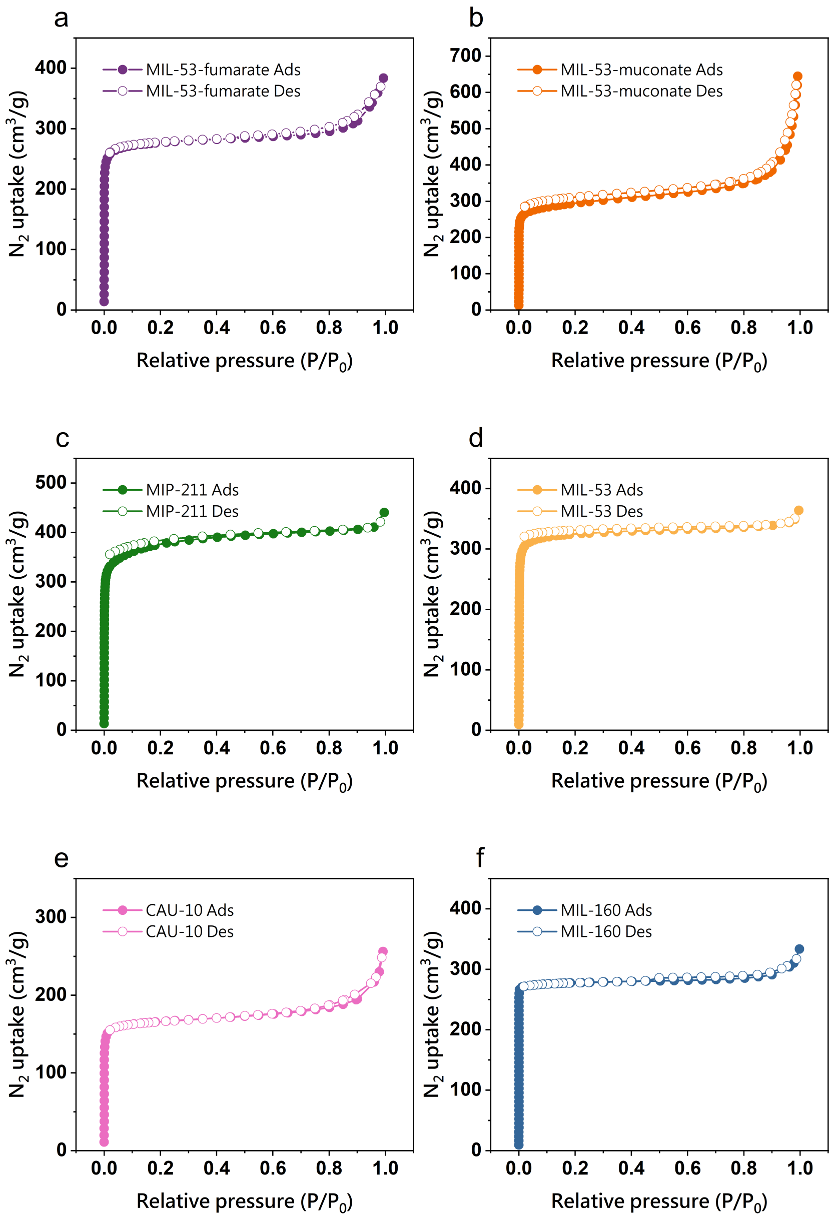

SI Fig. 4: N_2 sorption isotherms for a) MIL-53(Al)-muconate, b) MIL-53(Al), c) MIL-53(Al)-fumarate, d) MIP-211(Al), e) CAU-10(Al), f) MIL-160(Al).

Belsorp Adsorption/Desorption Data Analysis Software - Ver 6.4.1.0 MicrotracBEL Corp.

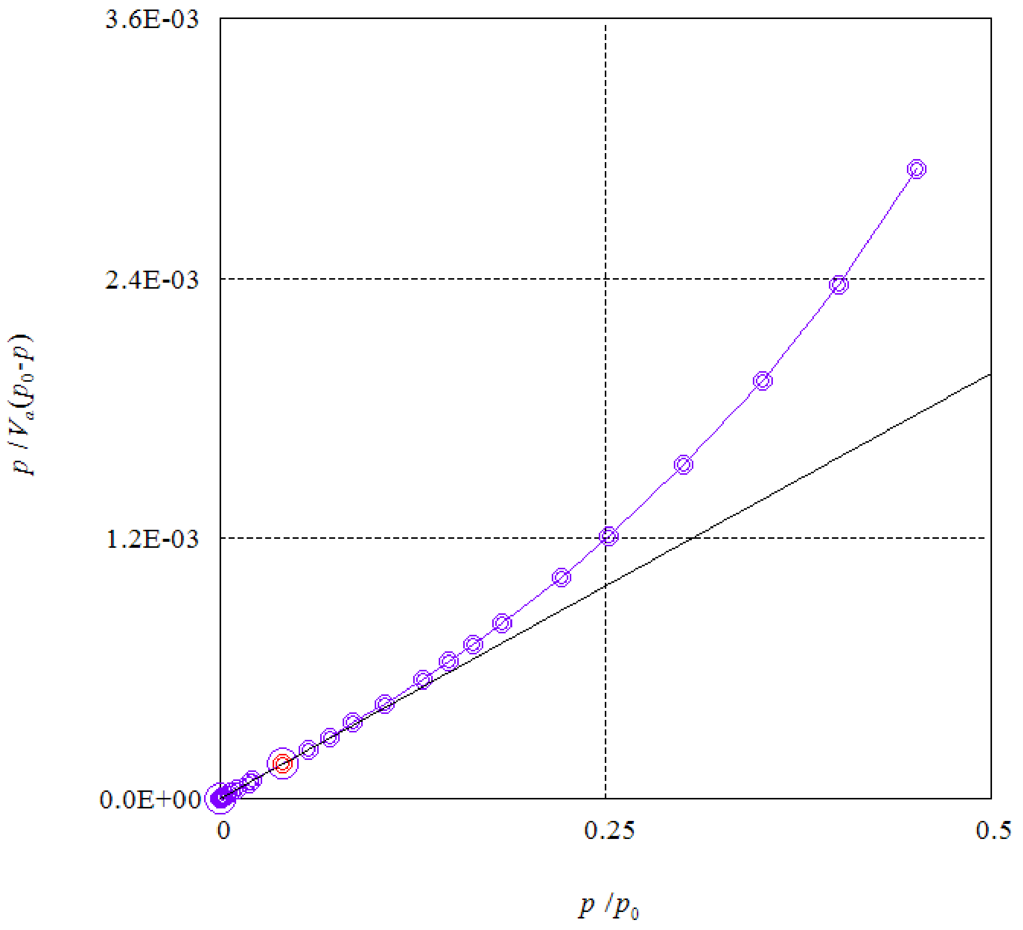

BET-Plot(Type I)

Adsorptive N2

Adsorption temperature77.000[K]

BET-MIL-53-fumarate.DAT

MIL-53-cryst.synt
LSMO
150C-12h
Leak amount -0.196Pa/min

Sample weight	4.7000E-02	[g]	Date of measurement	24/01/19
Saturated vapor pressure	97.891	[kPa]	Time of measurement	14:39:03

BET range limit	25 point number (p/p_0=4.0392E-02)			
$V_a(p_0-p)$Max value	2.4811E+04		V_m	255.12 [cm^3(STP) g^{-1}]
$a_{s,BET}$	1.1104E+03	[m^2 g^{-1}]	C	4150.6
Total pore volume(p/p_0=0.990)	0.5870	[cm^3 g^{-1}]	Mean pore diameter	2.1146 [nm]

Belsorp Adsorption/Desorption Data Analysis Software - Ver 6.4.1.0 MicrotracBEL Corp.

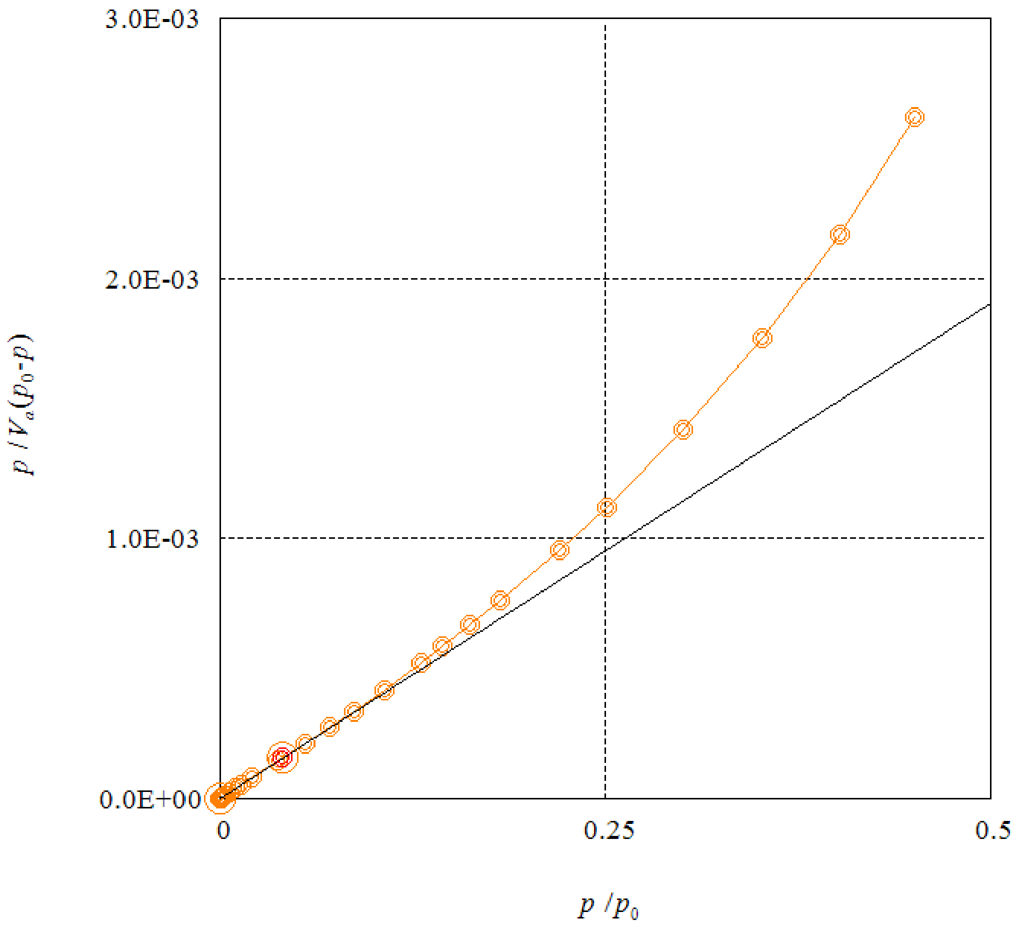

BET-Plot(Type I)

Adsorptive N2

Adsorption temperature77.000[K]

BET-MIL-53-muconate.DAT

MIL-53-muc
LSMO
Activated 175C 7h
Leak amount -0.196Pa/min

Sample weight	5.2000E-02	[g]	Date of measurement	25/03/29
Saturated vapor pressure	96.799	[kPa]	Time of measurement	26:44:15

BET range limit	29 point number (p/p_0=4.0843E-02)				
$V_a(p_0-p)$Max value	2.5306E+04		V_m	262.83	[cm^3(STP) g^{-1}]
$a_{s,BET}$	1.1440E+03	[m^2 g^{-1}]	C	4120.6	
Total pore volume(p/p_0=0.990)	0.9595	[cm^3 g^{-1}]	Mean pore diameter	3.3550	[nm]

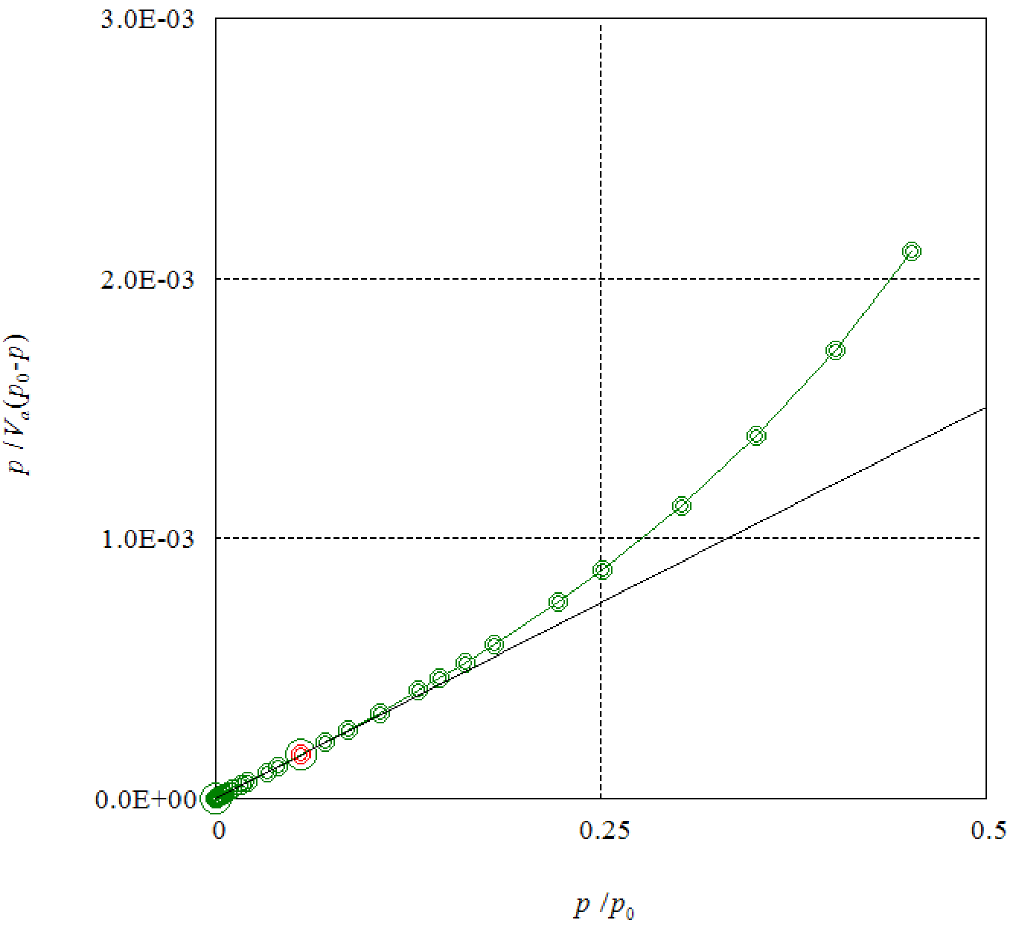

BET-Plot(Type I)

Adsorptive N2

Adsorption temperature77.000[K]

◎ BET-MIP-211.DAT

MIP-211
LSMO
Activated 175C 7h
Leak amount 0.684Pa/min

Sample weight	5.0000E-02	[g]	Date of measurement	25/04/03
Saturated vapor pressure	97.171	[kPa]	Time of measurement	31:07:23

BET range limit	39 point number (p/p_0=5.5089E-02)				
$V_a(p_0-p)$Max value	3.2036E+04		V_m	332.24	[cm^3(STP) g^{-1}]
$a_{s,BET}$	1.4461E+03	[m^2 g^{-1}]	C	2077.9	
Total pore volume(p/p_0=0.990)	0.6738	[cm^3 g^{-1}]	Mean pore diameter	1.8637	[nm]

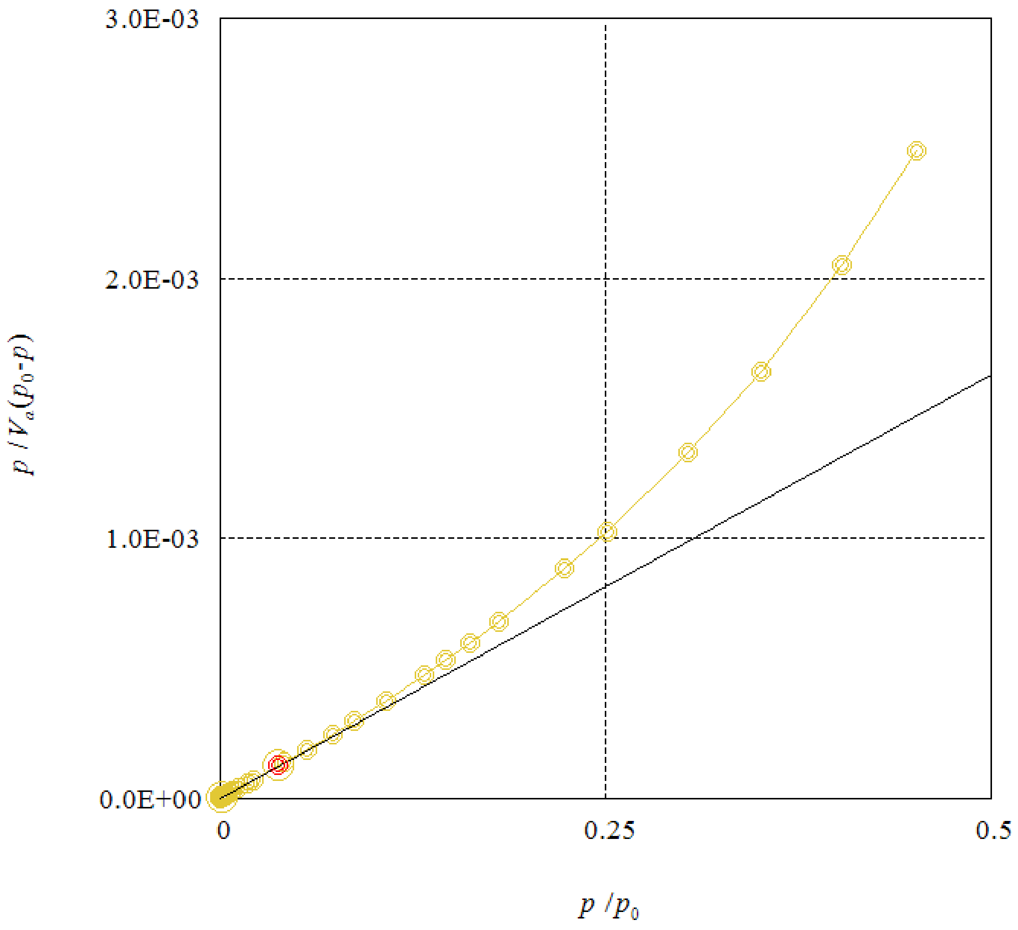

BET-Plot(Type I)

Adsorptive N2

Adsorption temperature77.000[K]

BET-MIL-53-Al.DAT

MIL-53'Al
LSMO
Activated 150C 12h
Leak amount 0.467Pa/min

Sample weight	6.7000E-02	[g]	Date of measurement	25/04/01
Saturated vapor pressure	96.706	[kPa]	Time of measurement	24:46:14

BET range limit	46 point number (p/p_0=3.8041E-02)				
$V_a(p_0-p)$Max value	2.8974E+04		V_m	306.68	[cm^3(STP) g^{-1}]
$a_{s,BET}$	1.3348E+03	[m^2 g^{-1}]	C	1492.2	
Total pore volume(p/p_0=0.990)	0.5538	[cm^3 g^{-1}]	Mean pore diameter	1.6595	[nm]

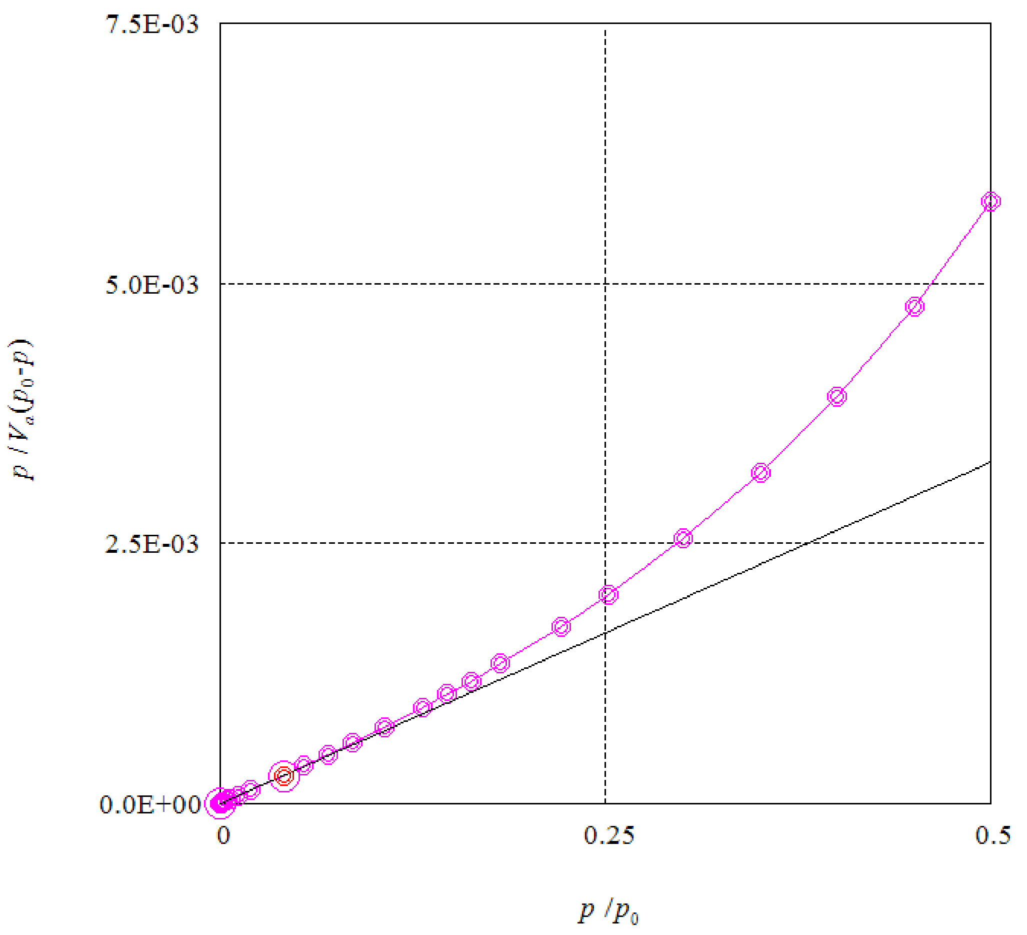

BET-Plot(Type I)

Adsorptive N2

Adsorption temperature77.000[K]

BET-CAU-10.DAT

CAU-10
LSMO
Activated 150C 12h
Leak amount -0.392Pa/min

Sample weight	6.3000E-02	[g]	Date of measurement	25/03/31
Saturated vapor pressure	97.176	[kPa]	Time of measurement	16:37:22

BET range limit	20 point number (p/p_0=4.1047E-02)			
$V_a(p_0\text{-}p)$Max value	1.4772E+04		V_m	152.66 [cm^3(STP) g^{-1}]
$a_{s,BET}$	6.6444E+02	[m^2 g^{-1}]	C	4583.2
Total pore volume(p/p_0=0.990)	0.3915	[cm^3 g^{-1}]	Mean pore diameter	2.3570 [nm]

Belsorp Adsorption/Desorption Data Analysis Software - Ver 6.4.1.0 MicrotracBEL Corp.

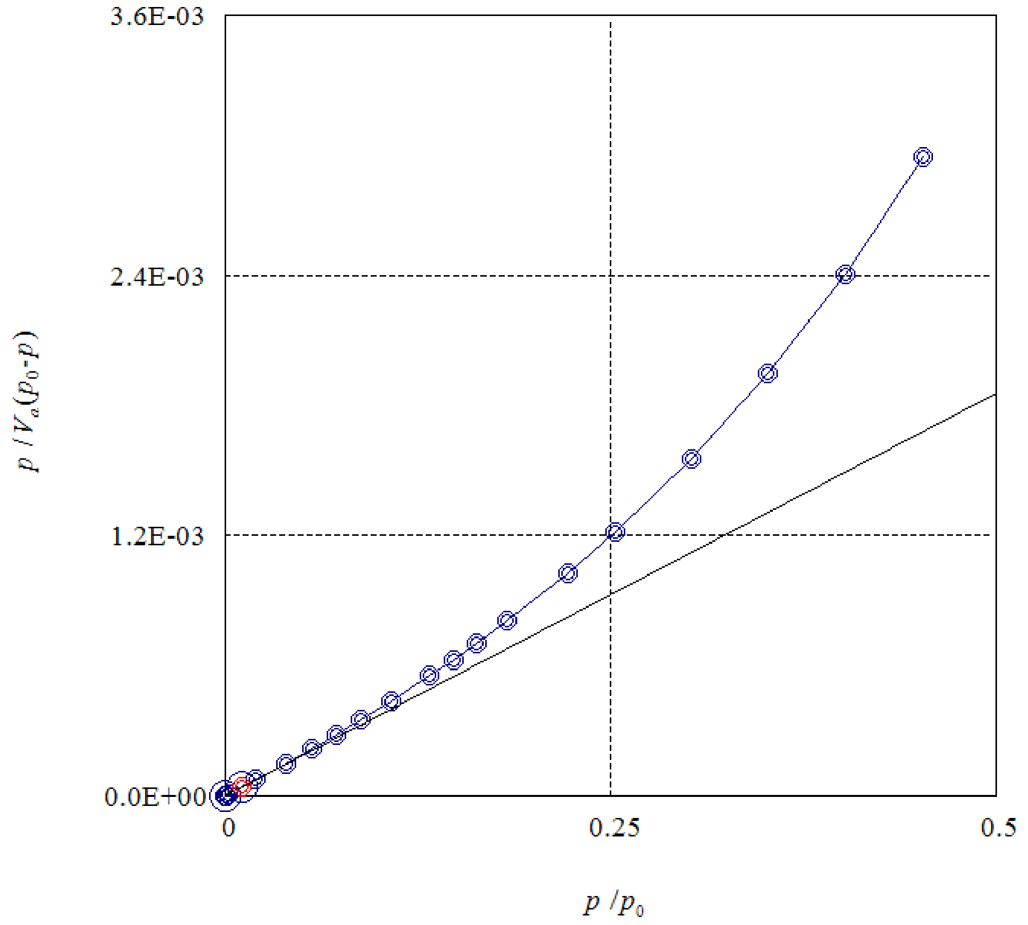

BET-Plot(Type I)

Adsorptive N2

Adsorption temperature77.000[K]

◎ BET-MIL-160.DAT

MIL-160
LSMO
Activated 150C 12h
Leak amount -0.315Pa/min

Sample weight	7.4000E-02	[g]	Date of measurement	25/03/31
Saturated vapor pressure	97.173	[kPa]	Time of measurement	17:40:31

BET range limit	38 point number (p/p_0=1.0845E-02)				
$V_a(p_0-p)$Max value	2.6014E+04		V_m	268.94	[cm^3(STP) g^{-1}]
$a_{s,BET}$	1.1706E+03	[m^2 g^{-1}]	C	2.0046E+04	
Total pore volume(p/p_0=0.990)	0.5022	[cm^3 g^{-1}]	Mean pore diameter	1.7160	[nm]

SI Fig. 5: BET data analysis for MIL-53(Al)-fumarate, MIL-53(Al)-muconate, MIP-211(Al), MIL-53(Al), CAU-10(Al), MIL-160(Al).

SI Table 3: Surface area and pore diameter of the synthesized MOFs.

MOF	Surface area ($m^2 g^{-1}$)	Pore diameter (Å)
MIP-211(Al)	1446	12
MIL-53(Al)	1335	9-11
MIL-53(Al)-muconate	1144	9.5-11
MIL-53(Al)-fumarate	1110	8.5
MIL-160(Al)	1170	7.8
CAU-10(Al)	664	8.5

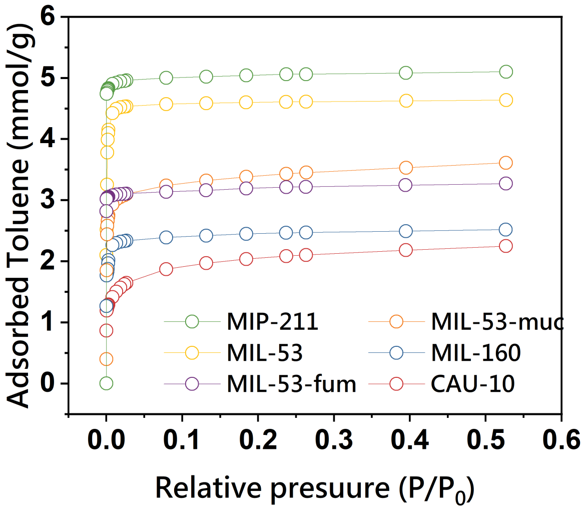

SI Fig. 6: Toluene desorption isotherms for MIP-211(Al), MIL-53(Al), MIL-53(Al)-fumarate, MIL-53(Al)-muconate, MIL-160(Al) and CAU-10(Al).

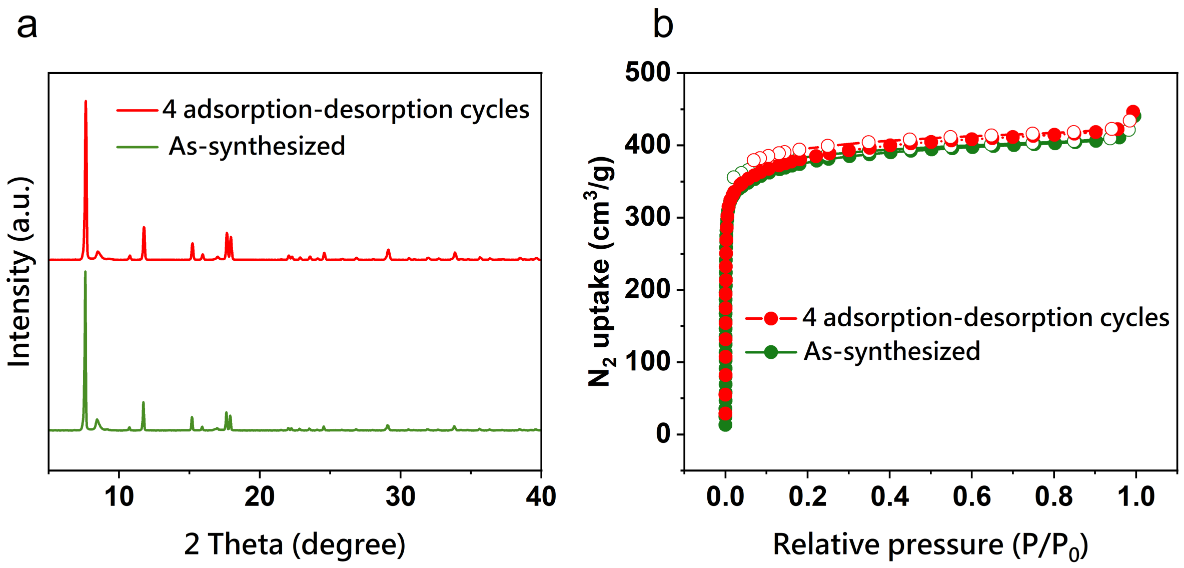

SI Fig. 7: a) Powder x-ray diffraction patterns of MIP-211(Al) before and after four toluene adsorption-desorption cycles using Cu Kα radiation (λ = 1.5418 Å), b) N_2 adsorption-desorption isotherms of MIP-211(Al) before and after four toluene adsorption-desorption cycles.

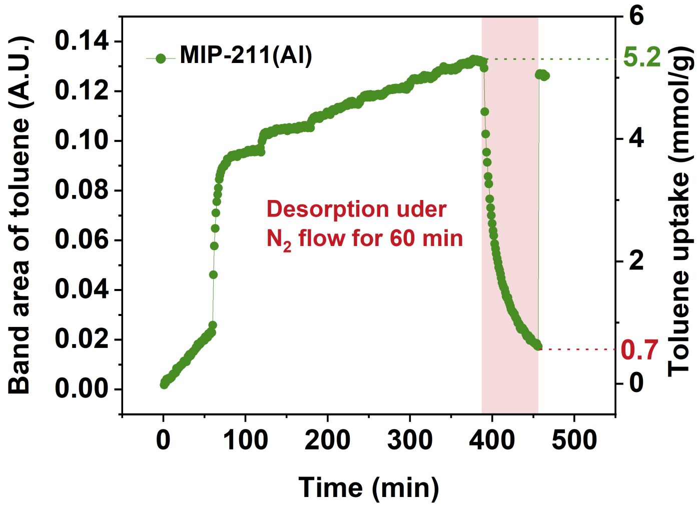

SI Fig. 8: Toluene adsorption/desorption of MIP-211(Al) recorded using ATR-FTIR spectroscopy. Desorption was performed under N_2 flow of 400 ml/min.

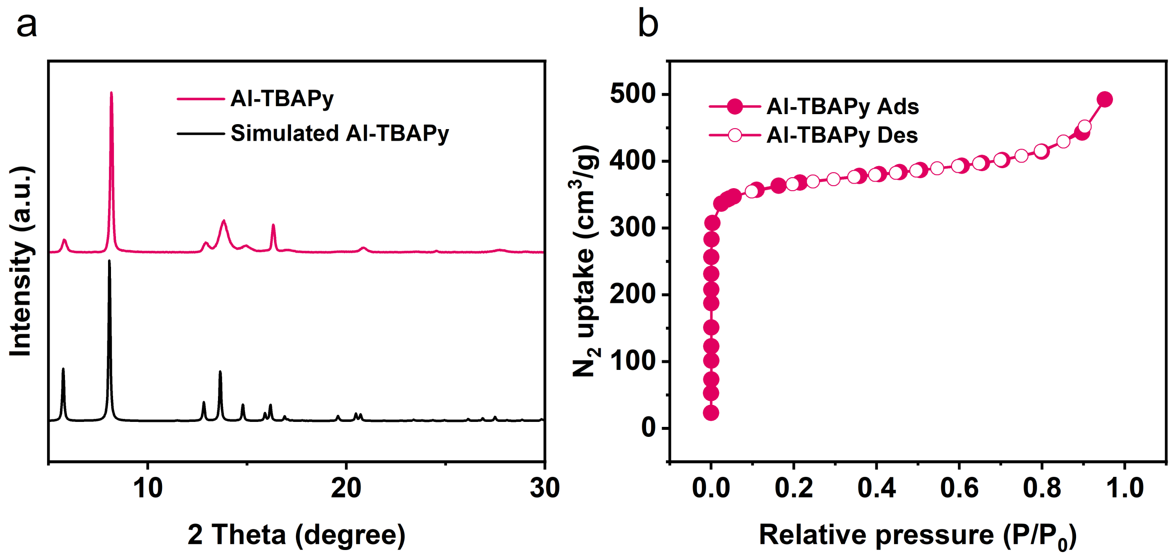

SI Fig. 9: a) Experimental powder x-ray diffraction patterns using Cu Kα radiation (λ = 1.5418 Å) in comparison with simulated for Al-TBAPy, b) N_2 sorption isotherms for Al-TBAPy

SI Table 4: Toluene uptake (mmol g^{-1}) of various MOFs at different relative pressures (P/P_0) recorded by static sorption analysis.

MOF	0.0026	0.01	0.1	Reference
HKUST-1(Cu)	3.6	4.6	5.4	[31]
ZJU-520(Al)	0.5 (P/P_0=0.006)	0.65	6	[5]
ZJU-620(Al)	1.5	3.3	3.5	[32]
BUT-66(Zr)	0.007	0.7	1.9	[33]
CAU-1(Al)	0.23	1.08	1.8	[15]
CAU-10(Al)	1.24	1.47	1.96	this work
MIL-53(Al)	1.81	4.2	4.55	this work
MIL-160(Al)	1.83	2.28	2.41	this work
MIL-101(Cr)	–	2	9	[7]
Activated carbon	3.2	3.6	4	[34]
DUT-4(Al)	3.16	4.34	–	[14]
MIP-211(Al)	4.8	4.92	5	this work
MIL-53(Al)-muconate	2.65	3.22	3.23	this work
MIL-53(Al)-fumarate	2.99	3.05	3.11	this work
MOF-74(Co)	–	–	0.87	[35]
MIL-100(Fe)	1.63	3.26	4.88	[36]
MIL-101(Fe)	–	–	0.65	[36]
MIL-53(Fe)	–	–	0.001	[36]
UiO-66(Zr)	1.76	2.33	2.64	[13]
UiO-66(Zr)-defects	2.11	3.52	4.09	[13]
Ga-BPTp(Ga)	0.5	6.7	7.5	[8]

S2 Molecular simulation details

S2.1 MOF Database

The MOFs structures come from the Prisma datasets,[37] in which all structures are optimized using density functional theory (DFT) calculation. The DFT calculations were performed using the Perdew–Burke–Ernzerhof (PBE) functional within the generalized gradient approximation (GGA),[38] with van der Waals (vdW) interactions included through Grimme's D3 corrections.[39]

S2.2 Henry coefficient calculation

The henry coefficients are obtained from Grand Canonical Monte Carlo (GCMC) simulations performed in RASPA software.[40] 100000 cycles of widom insetions were done by the *Isotherm* work chain in the *SinglecompWidom* plugin.[41–43]

During the simulations, we considered van der Waals and electrostatic interactions to describe the energy surface, represented respectively by the Lennard-Jones (LJ) potential and Coulomb interactions. Periodic boundary conditions were employed with a cutoff radius of 12.8 Å, including tail corrections to remedy the truncation. Density-derived electrostatic and chemical (DDEC) method is used to compute the partial charges on the atoms of the MOF frameworks.[44] The Ewald summation technique was used to model Coulomb interaction. The dispersion interactions of the framework and the gases were modeled with Lennard-Jones potentials. The toluene (10 sites) force field in TraPPE[45,46] is used to model the toluene-toluene interactions. The Lennard-Jones parameters for all MOFs were used from the Universal Force Field (UFF).[47]

S2.3 Isotherm generation

The toluene isotherms are obtained from GCMC simulations, performed in RASPA.[40] 15000 cycles were used for equilibration and 15000 cycles for production. Simulations at subsequent pressure points were performed starting from the restart file of the previous pressure step, thus reducing the number of cycles necessary for initialization. The process

was done by the *Isotherm* work chain in the *aiida-lsmo* plugin.[41]

Li *et al.*[48] developed a refined force field for MOFs containing group IIA metals (Mg, Ca, Sr, and Ba) and group IIIA metals (Al, Ga, and In), as the UFF systematically overestimates adsorption in these systems. Accordingly, we applied this refined UFF to MOFs containing these metals, while the UFF was used for all other MOFs. Toluene adsorption isotherms were then simulated and validated with experimental data below.

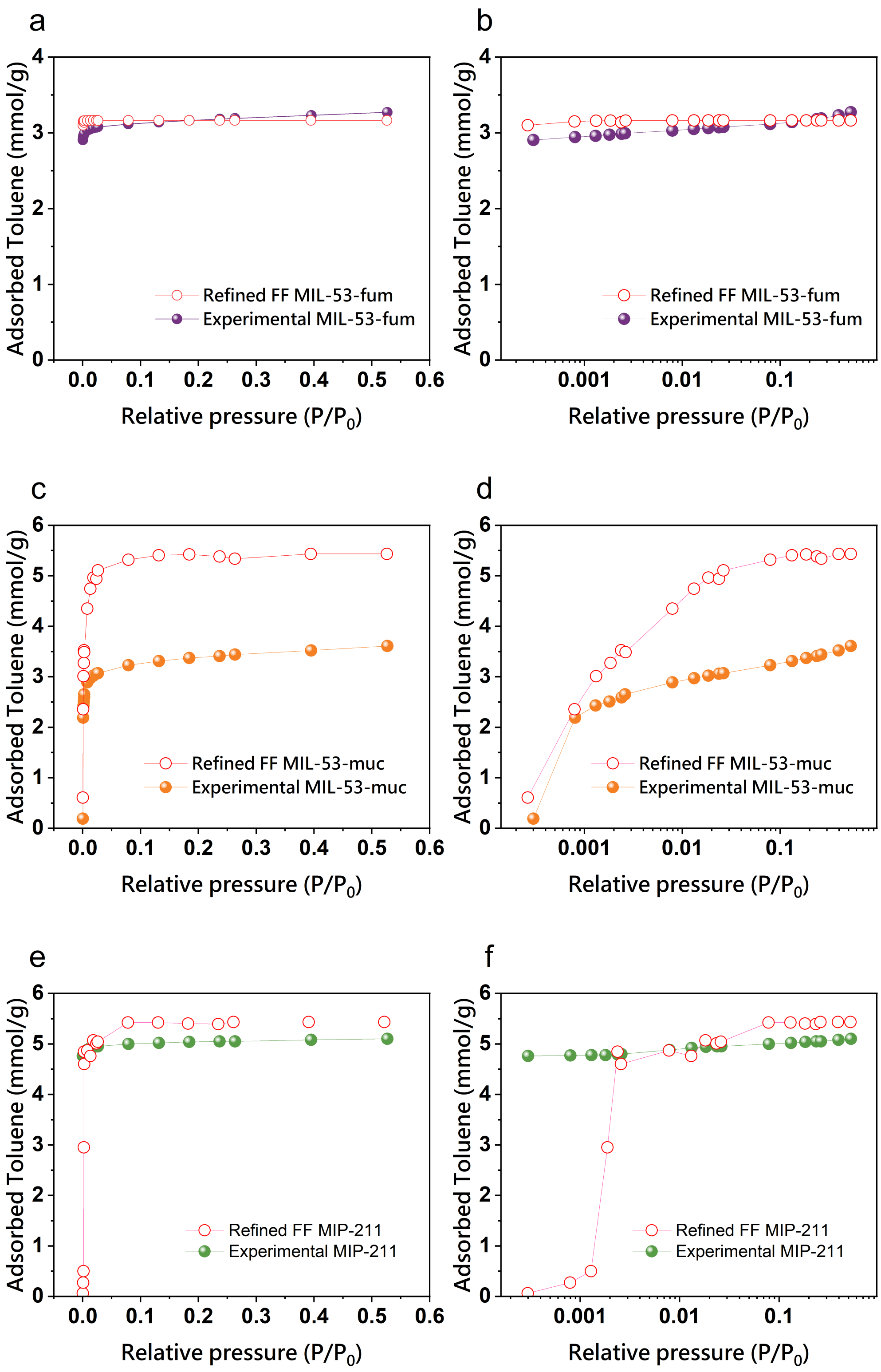

SI Fig. 10: Toluene isotherms experimental versus simulated using refined force field (FF) at linear and logarithmic scale for a),b) MIL-53(Al)-fumarate, c), d) MIL-53(Al)-muconate, e), f) MIP-211(Al).

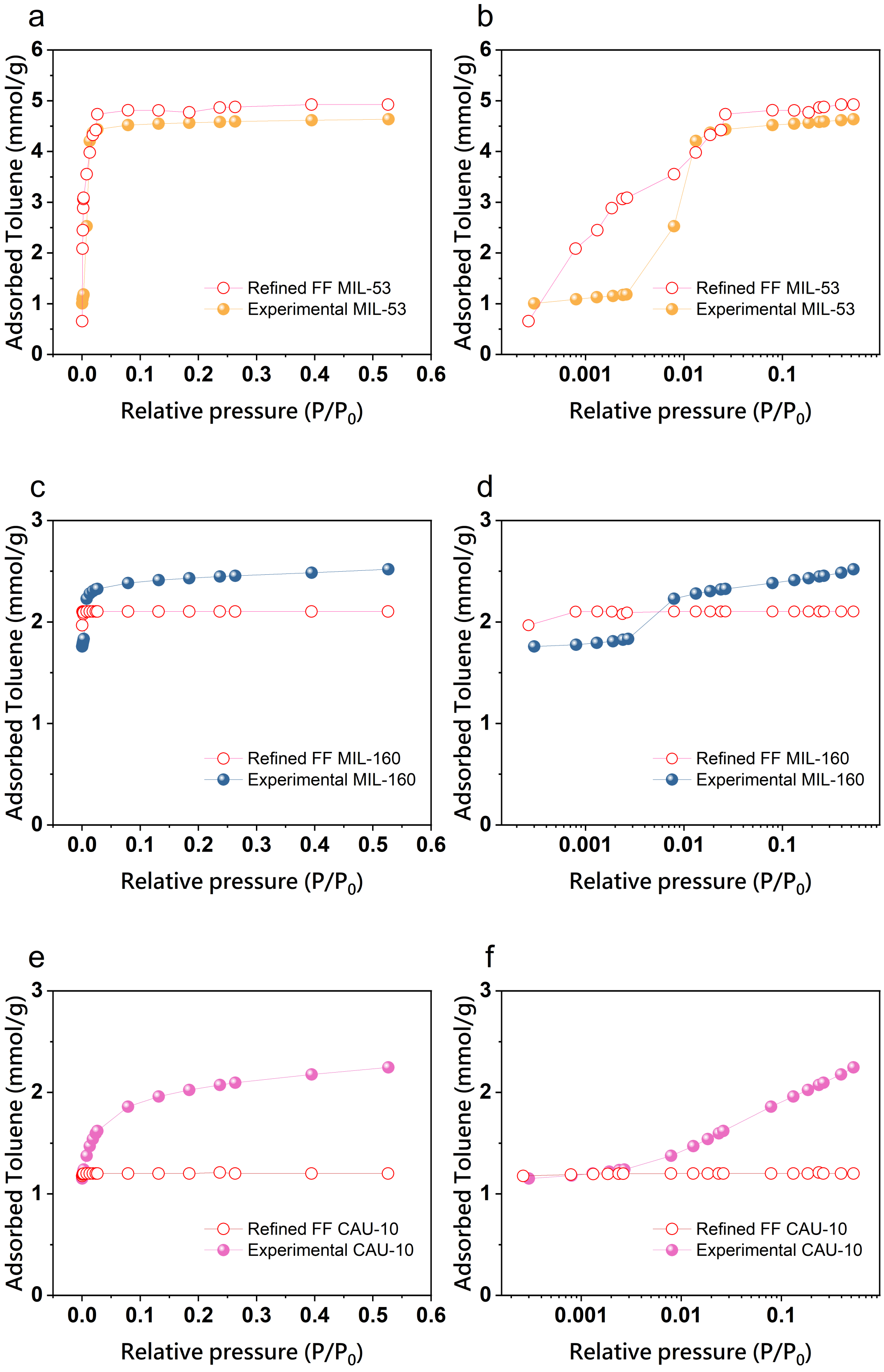

SI Fig. 11: Toluene isotherms experimental versus simulated using refined force field (FF) at linear and logarithmic scale for a),b) MIL-53(Al), c), d) MIL-160(Al), e), f) CAU-10(Al).

S2.4 Database refinement based on pore geometry

The initial database was optimized by selecting MOFs featuring only one-dimensional channels with quadrilateral cross-sections, as shown in Figure S 12(b). To achieve this goal, we used **Zeo**++software[49] to determine channel dimensionality and focused on M-MOFs (M = Al, In, Ga), which are known for their rod-metal chains and good water stability. As illustrated in Figure S 12(a), we then ranked these structures based on simulated toluene uptake and K_H for toluene.

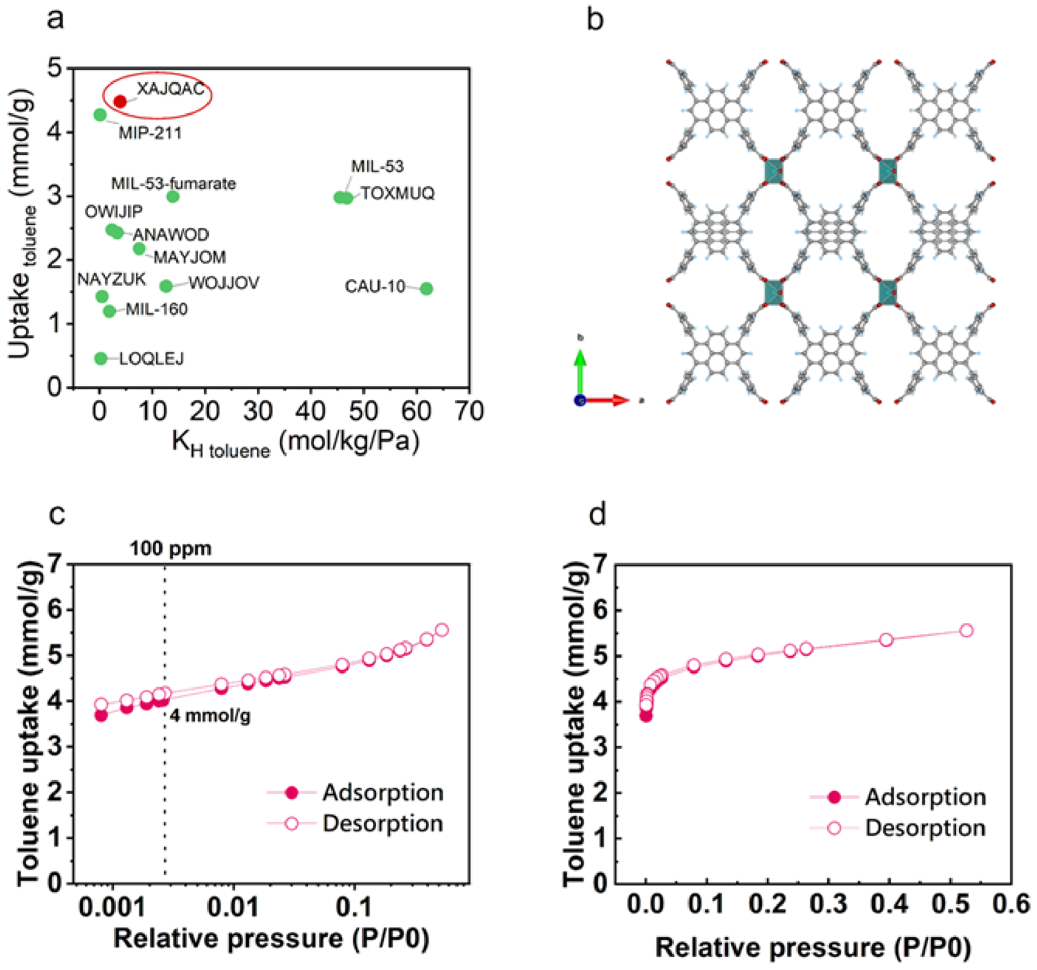

SI Fig. 12: a) Ranking of MOFs based on simulated toluene uptake at P/P_0=0.0026 and K_H for toluene; b) Crystal structure of Al-TBAPy ; c) Toluene isotherm for Al-TBAPy at logarithmic and d) linear scale of relative pressure.

S3 Synchrotron powder X-ray diffraction (SPXRD) experiment

Synchrotron powder X-ray diffraction experiment was conducted at BM01 from the Swiss-Norwegian Beamlines located at the European Synchrotron Radiation Facility (ESRF, Grenoble).

Sample preparation

MIP-211(Al) was activated at 175°C for 7 hours under vacuum and soaked in toluene for 10 min. The sample was filled up to 1 mm capillary and connected to the gas-loading system. The sample was heated using Cryostream 700+ from 300 to 500 K with 6 K/min rate under dynamic vacuum and the 2D diffraction images were collected every 20 seconds.

To investigate the structural response of MIP-211(Al) to the toluene sorption and desorption,we subjected the toluene-loaded MOF to a controlled thermal treatment under dynamic vacuum, following a temperature regime of 300K $\rightarrow$ 500K $\rightarrow$ 300K. During the heating process, significant changes in relative peak intensities were observed, indicating the release of toluene molecules from the framework Figure S 13.

Le Bail refinement enabled determination of the unit cell parameters throughout the thermal cycle Figure S 16a. As shown in Figure S 16b, the unit cell volume initially increases between 300K and 330K due to thermal expansion. Further heating results in a slight contraction, which likely reflects the competing effects of framework expansion and partial toluene desorption. Continued heating results in a steady increase in unit cell volume, suggesting complete evacuation of toluene by 400K and structural stabilization at 500K. Upon cooling back to 300K, the unit cell volume of the evacuated framework remains slightly larger than that of the toluene-loaded form, revealing a contraction of the framework upon toluene adsorption. Notably, both lattice parameters, $\boldsymbol{a}$ and $\boldsymbol{c}$, exhibit reductions of 0.06% and 0.05%, respectively Figure S 16c,d, confirming the subtle framework shrinkage associated with toluene uptake.

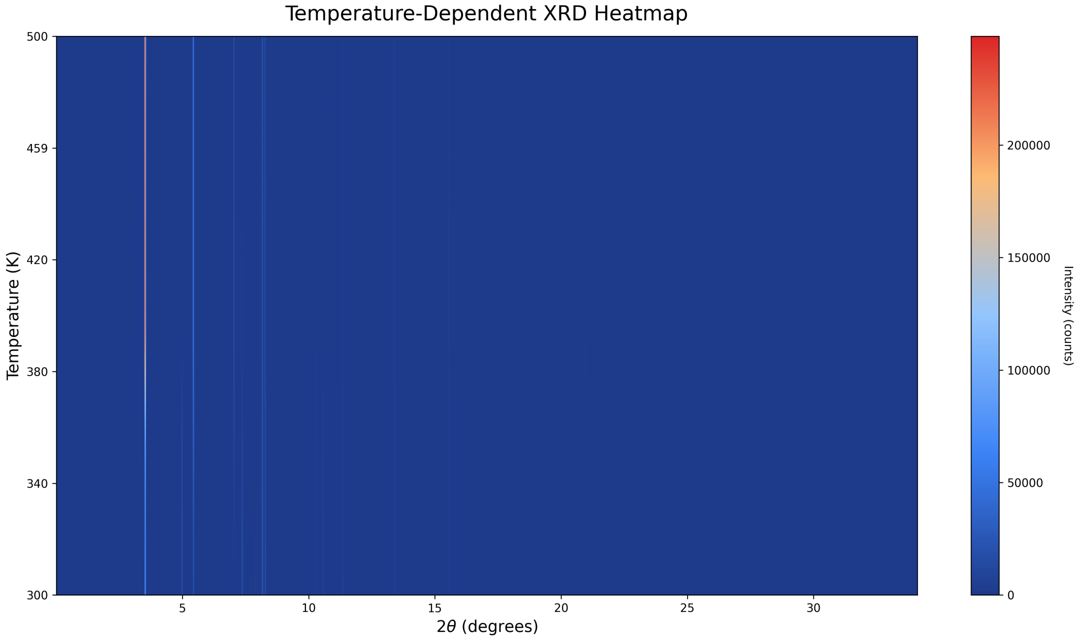

SI Fig. 13: SPXRD variable-temperature plot, showing the changes of relative intensity of peaks for MIP-211(Al) loaded with toluene.

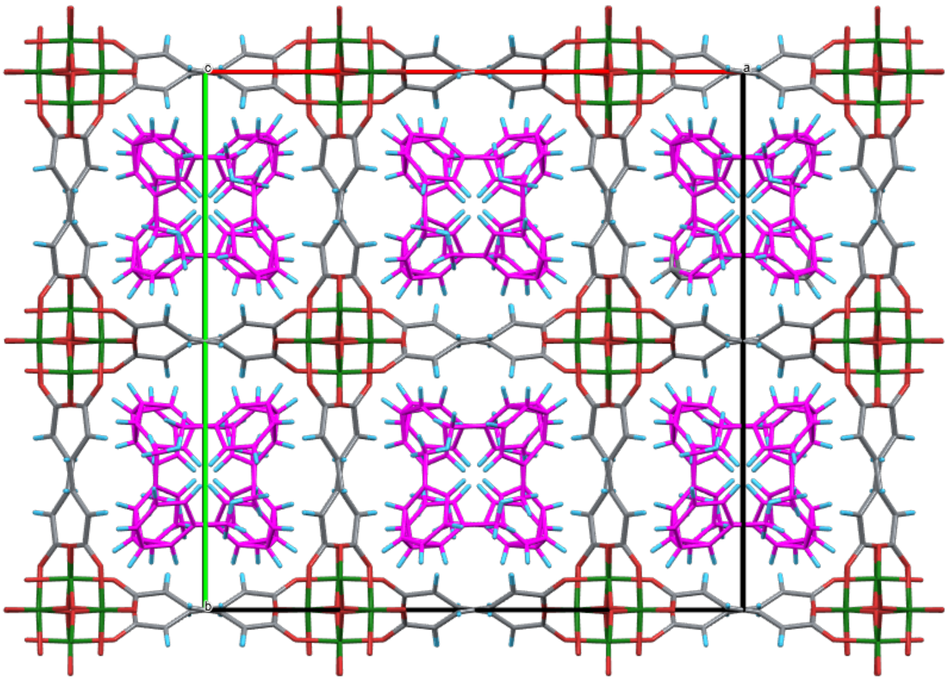

SI Fig. 14: The experimental toluene positions in MIP-211(Al) according to crystal structure refinement using the Rietveld method. The position of molecule is filled by $\frac{1}{2}$. Carbon atoms of toluene are marked by pink color.

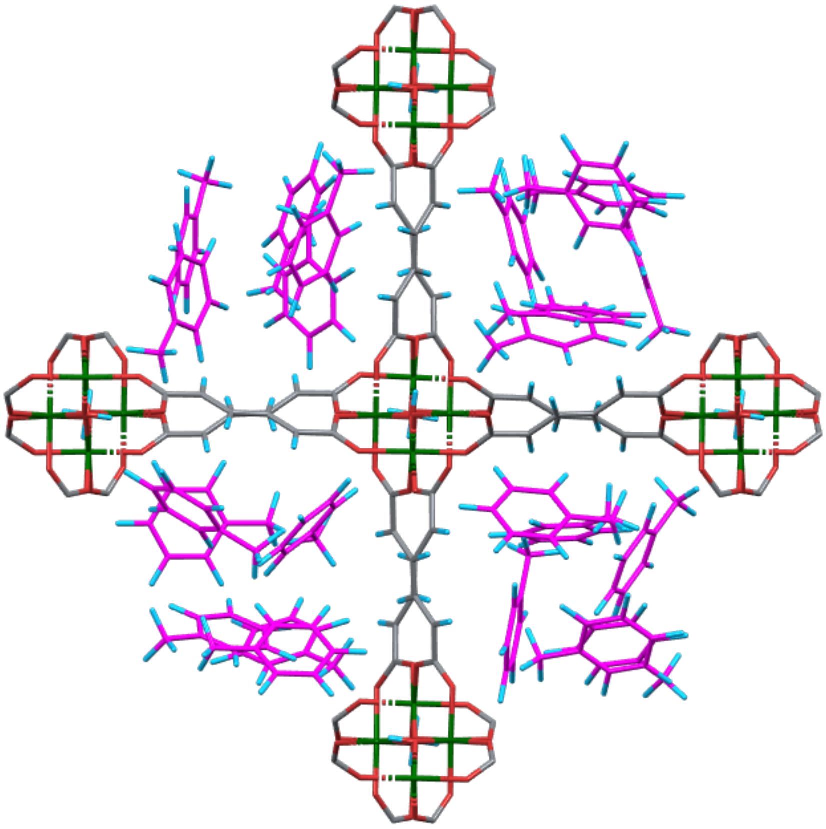

SI Fig. 15: Toluene positions in MIP-211(Al) according DFT simulations. Carbon atoms of toluene are marked by pink color.

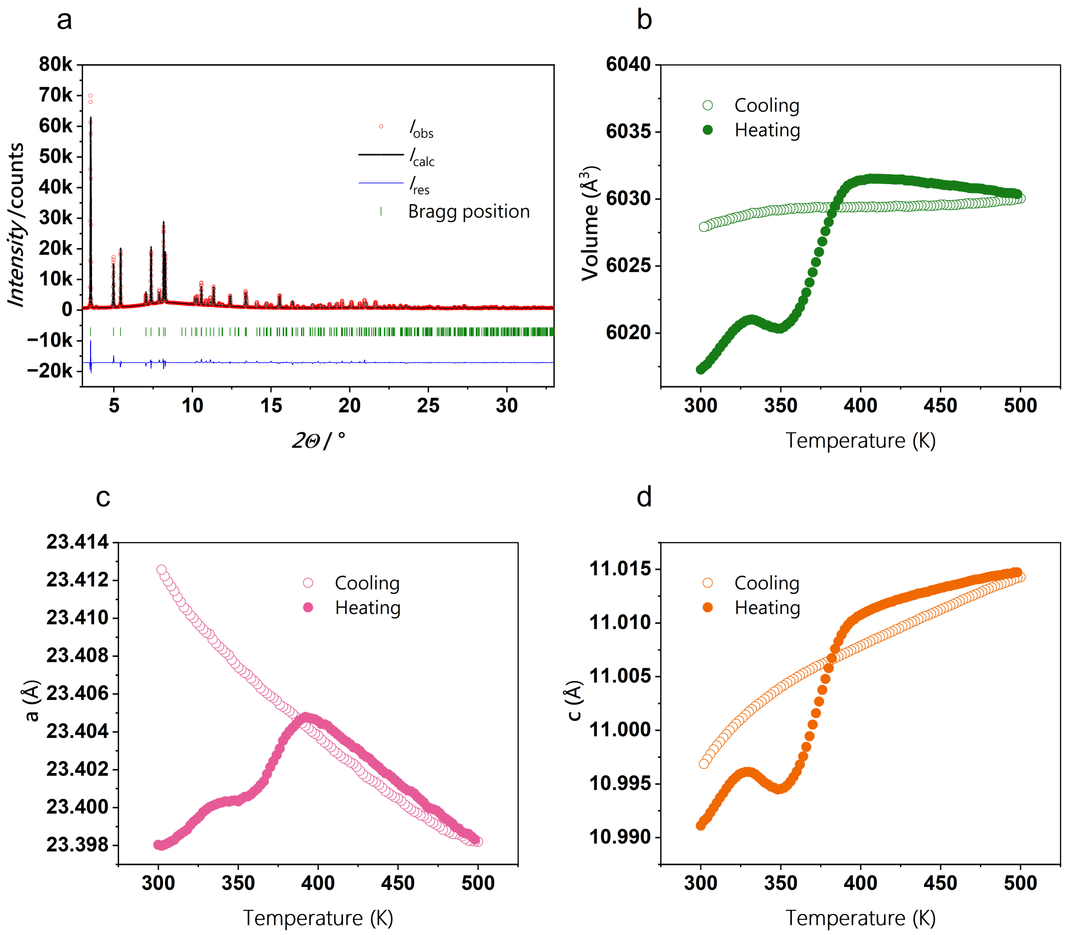

SI Fig. 16: (a) Rietveld refinement plot of MIP-211(Al) loaded with toluene (space group $I4_1/acd$: $a = b = 23.4089(2)$ Å, $c = 11.0037(2)$ Å, $R_I = 8.46\%$, $R_F = 8.45\%$, $\lambda = 0.71813$ Å), (b) Temperature-dependent changes in unit cell volume; (c) Variation of lattice parameter a with temperature; (d) Variation of lattice parameter c with temperature.

S4 ATR-FTIR spectroscopy

The MOF powder was dispersed in hexane and drop–casted onto an ATR crystal, which was fabricated from a low-resistivity, double-side polished Si wafer ($20 \times 10 \times 0.5 mm^3$, 45°). For toluene sorption measurements, the ATR crystal was placed into a custom-made mount and sealed using an FKM O–ring and an aluminum gas flow cell with a volume of approximately 1 cm^3. Toluene and water vapors at various relative vapor pressures (P/P_0) were generated by mixing dry N_2 with N_2 saturated with toluene or water vapor *via* bubbling at room temperature, using mass flow controllers (Bronkhorst). The actual vapor concentrations were determined using transmission IR spectroscopy in a 10 cm transmission cell with ZnSe windows. Calibration was performed by integrating transmission spectra in the 1700–1800 cm^{-1} range for water and around 2800 cm^{-1} for toluene, using reference spectra (1 ppm/m) from the PNNL database.[50] Before starting the measurements, the spectrometer was flushed with N_2 for 10 minutes. During the experiments, the vapor pressures of $P/P_0 = 0.02$–0.07 were maintained for 60 minutes to allow equilibrium, while higher pressures ($P/P_0 = 0.12$–0.48) were held for 30 minutes. The main source of uncertainty arises from the ATR measurement step. While the adsorption coefficient obtained from KBr pellets is well defined, the MOF on the ATR crystal must be referenced to a separate blank ATR crystal. Because the blank and MOF–loaded ATR crystals cannot be positioned identically within in our setup, small differences in the background spectra introduce additional uncertainty in the MOF quantification.

S4.1 Quantification in ATR–FTIR spectroscopy

Lambert–Beer's law (Eq. S1) was applied for quantification using FTIR spectroscopy, where A represents the absorbance, ε the absorption coefficient, C the concentration, and d_e the effective path length:

$$A = \varepsilon \cdot C \cdot d_\mathrm{e} \tag{1}$$

According to Eq. S1, the amount of MOF present within the evanescent wave on the

ATR crystal can be found as:

$$C = \frac{A}{\varepsilon \cdot d_e} \tag{2}$$

S4.2 Determination of the MOF amount present on the ATR crystal

To calculate the volume of MOF deposited on the ATR crystal ($V_{\mathrm{MOF}_{\mathrm{ATR}}}$), we use Eq. S3.

$$V_{\mathrm{MOF}_{\mathrm{ATR}}} = V_E \cdot \phi_{\mathrm{MOF}} \tag{3}$$

Where V_{E} is the total evanescent field volume, that represents the volume of the flow cell region probed by the infrared radiation and is defined as:

$$V_{\mathrm{E}} = \pi \, r^2_{\text{O-ring}} \cdot d_e \tag{4}$$

using the known radius of the O-ring of the flow cell ($r_{\text{O-ring}} = 7$ mm) and a height corresponding to the total effective path length (d_{e}).

The fraction of MOF (ϕ_{MOF}) present in the evanescent field on the ATR crystal can be determined from the MOF absorbance of the C=O vibration at 1426 cm^{-1} on the ATR crystal (A_{ATR}), the absorption coefficient of ($\varepsilon_{\mathrm{MOF}}$), and the effective path length at at 1426 cm^{-1} (d_e) as:

$$\phi_{\mathrm{MOF}} = \frac{A_{\mathrm{ATR}}}{\varepsilon \cdot d_{\mathrm{e}}} \tag{5}$$

A_{ATR} was obtained from the band intensity of the C=O vibration at 1426 cm^{-1} of the MOF linker, using a blank ATR crystal spectrum as background.

d_{e} of the ATR crystal at 1426 cm^{-1} can be determined following the method proposed by Harrick and Du Pre[51] from the number of active reflections (N), obtained from a reference measurement of a blank ATR crystal, and equals 0.0013.

$\varepsilon_{\mathrm{MOF}}$ of MIP-211 was determined from KBr reference measurements using Eq. S6:

$$\varepsilon_{\mathrm{MOF}} = \frac{A}{c \cdot d_{\mathrm{e}}} = 8082\ \mathrm{cm}^{-1} \tag{6}$$

Blank and MIP-211(Al) containing KBr pellets were prepared by mixing 3.24 mg of MOF with 661.1 mg of KBr, 100 mg of this mixture was transferred into the pellet press. Reference FTIR spectra were recorded in transmission mode. A was obtained from the band intensity of the C=O vibration at 1426 cm^{-1} of the MOF linker, using a blank KBr pellet spectrum as background Figure S 18a. The path length (d_{e}) was defined as the thickness of the pellet and calculated from the known pellet mass ($m_{\mathrm{KBr}} = 0.0964g$), pellet radius ($r = 6,5mm$), and the density of KBr ($\rho_{\mathrm{KBr}} = 2.75\ \mathrm{g\,cm}^{-3}$) using Eq. S7:

$$V_{\mathrm{KBr}} = \pi r^2 d_{\mathrm{e}} = \frac{m_{\mathrm{KBr}}}{\rho_{\mathrm{KBr}}} \iff d_{\mathrm{e}} = \frac{m_{\mathrm{KBr}}}{\rho_{\mathrm{KBr}} \cdot \pi r^2} \tag{7}$$

S4.3 Determination of the toluene amount absorbed by MOF

Toluene concentration in $\mathrm{mol\,cm}^{-3}$ can be calculated using Eq. S8:

$$C_{\mathrm{toluene}} = \frac{A_{\mathrm{area}}}{\varepsilon_{\mathrm{toluene}} \cdot V_{\mathrm{e}} \cdot 1000} \tag{8}$$

Therefore amount of toluene in mol n_{toluene} Eq. S9:

$$n_{\mathrm{toluene}} = \frac{A_{\mathrm{area}}}{\varepsilon_{\mathrm{toluene}} \cdot 1000} \tag{9}$$

As a result, the toluene uptake in $\mathrm{mmol\,g}^{-1}$ can determined using skeletal density of the MOF (for MIP-211 ρ_{skeletal}=2,2635 $\mathrm{g\,cm}^{-3}$ according to gas pycnometry) Eq. S10:

$$Uptake = \frac{n_{\mathrm{toluene}} \cdot 1000}{V_{\mathrm{MOF_{ATR}}} \cdot \rho_{\mathrm{skeletal}}} \tag{10}$$

S4.4 Determination of the water amount absorbed by MOF

Water uptake in the MOF was determined by normalizing the maximum band area of the water signal using reference values from the water sorption isotherm (SI Fig. 16).

The corresponding uptake values were 3.4 $\mathrm{mmol\,g^{-1}}$ at 15% relative humidity (RH) and 30.8 $\mathrm{mmol\,g^{-1}}$ at 40% RH.

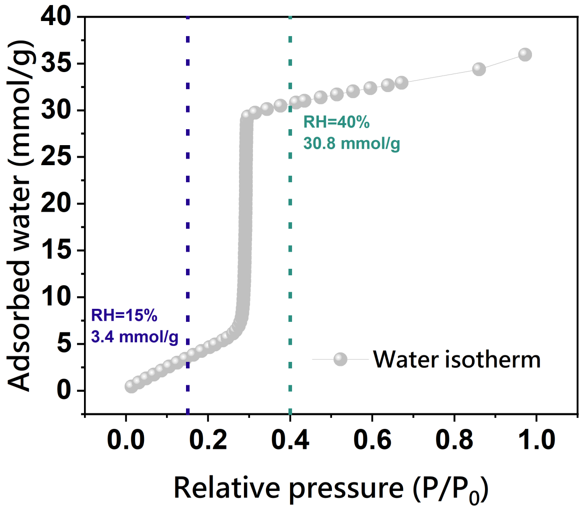

SI Fig. 17: Water sorption isotherm for MIP-211(Al).

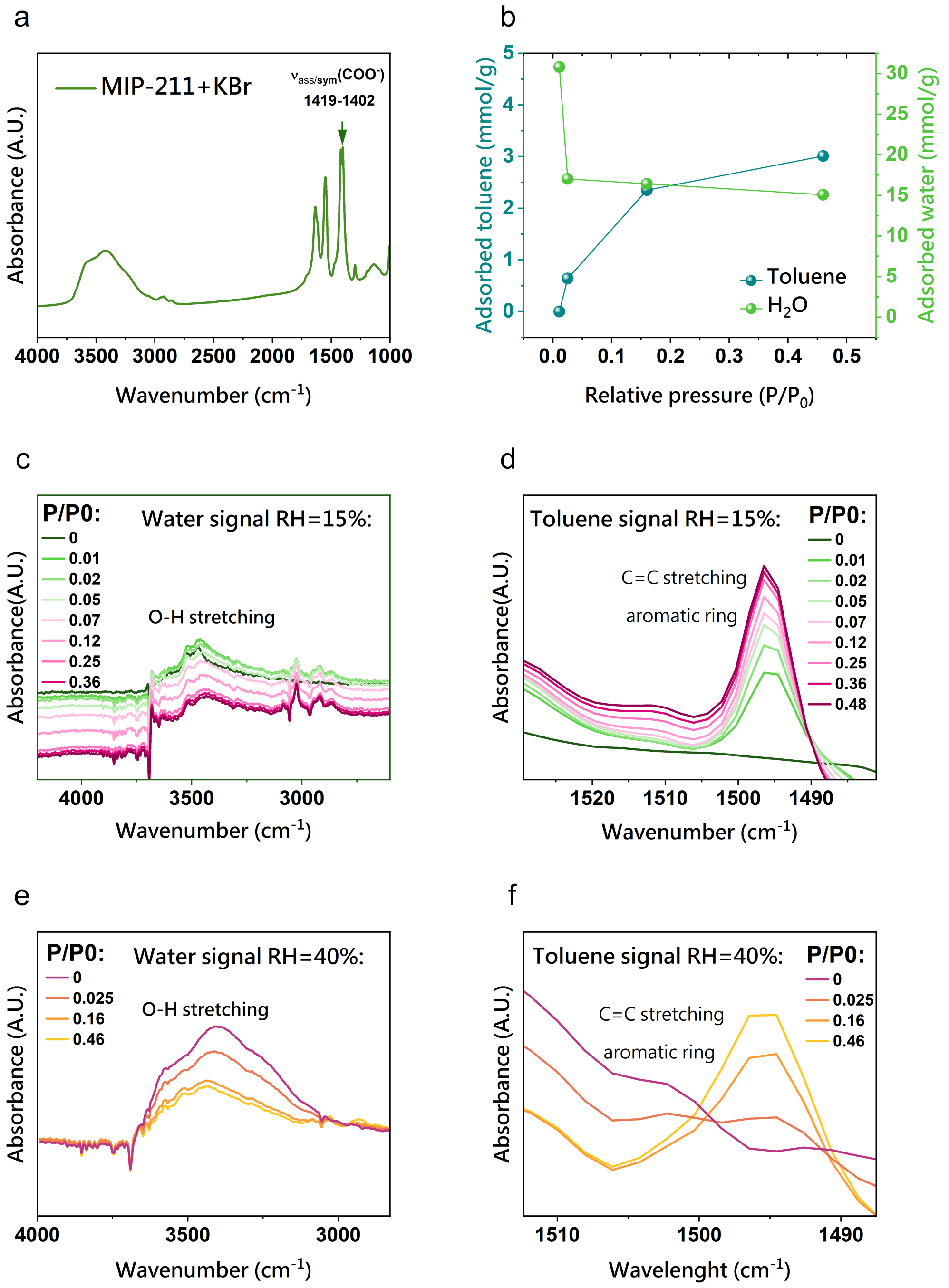

SI Fig. 18: a) FTIR spectra of MIP-211(Al) mixed with KBr; b) ATR-FTIR toluene isotherms at RH = 40% versus water uptake; c) FTIR spectra of water signal in MIP-211(Al) at RH = 15% and different partial pressures of toluene; d) FTIR spectra of toluene signal in MIP-211(Al) at RH = 15% and different partial pressures of toluene; e) FTIR spectra of water signal in MIP-211(Al) at RH = 40% and different partial pressures of toluene; f) FTIR spectra of toluene signal in MIP-211(Al) at RH = 40% and different partial pressures of toluene.

References

(1) Jhung, S. H.; Lee, J.-H. .; Yoon, J. W. .; Serre, C. .; Férey, G. .; Chang, J.-S. Microwave Synthesis of Chromium Terephthalate MIL-101 and Its Benzene Sorption Ability. *Adv. Mater.* **2007**, *19*, 121.

(2) Zhang, X.; Wang, Y. .; Mi, J.; Jin, J.; Meng, H. Dual Hydrophobic Modification on MIL-101 (Cr) with Outstanding Toluene Removal Under High Relative Humidity. *Chem. Eng. J.* **2023**, *451*, 139000.

(3) Gwardiak, S.; Szczęśniak, B. .; Choma, J. .; Jaroniec, M. Benzene Adsorption on Synthesized and Commercial Metal–Organic Frameworks. *J. Porous Mater.* **2019**, *26*, 775–783.

(4) Lv, J.-A.; Tang, Z.-L. .; Liu, Y.-H. .; Zhao, R.-C.; Xie, L.-H.; Liu, X.-M.; Li, J.-R. Interior and Exterior Surface Modification of Zr-Based Metal–Organic Frameworks for Trace Benzene Removal. *Inorg. Chem.* **2024**, *63*, 4249–4259.

(5) Hu, L.; Wu, W. .; Hu, M. .; Jiang, L.; Lin, D.; Wu, J. .; Yang, K. Double-Walled Al-Based MOF with Large Microporous Specific Surface Area for Trace Benzene Adsorption. *Nat. Commun.* **2024**, *15*, 3204.

(6) Zhao, Z.; Wang, S. .; Yang, Y. .; Li, X.; Li, J. .; Li, Z. Competitive Adsorption and Selectivity of Benzene and Water Vapor on the Microporous Metal Organic Frameworks (HKUST-1). *Chem. Eng. J.* **2015**, *259*, 79–89.

(7) Xu, F.; Xian, S.; Xia, Q. .; Li, Y.; Li, Z. Effect of Textural Properties on the Adsorption and Desorption of Toluene on the Metal-Organic Frameworks HKUST-1 and MIL-101. *Adsorp. Sci. Technol.* **2013**, *31*, 325–339.

(8) Tu, T. N.; Kwon, H. T.; Scheer, M.; Kim, J. High Toluene Uptake at a Trace Concentration in a Novel Gallium-Based Metal–Organic Framework. *J. Mater. Chem. A* **2025**, *13*, 9479–9485.

(9) Han, Y.; Huang, W.; He, M.; An, B.; Chen, Y.; Han, X.; An, L.; Kippax-Jones, M.; Li, J.; Yang, Y., et al. Trace Benzene Capture by Decoration of Structural Defects in Metal–Organic Framework Materials. *Nat. Mater.* **2024**, *23*, 1531–1538.

(10) Han, Y.; Chen, Y. .; Ma, Y. .; Bailey, J. .; Wang, Z. ., et al. Control of the Pore Chemistry in Metal-Organic Frameworks for Efficient Adsorption of Benzene and Separation of Benzene/Cyclohexane. *Chem. Rev.* **2010**, *110*, 5989–6008.

(11) Han, Y.; Brooks, D. .; He, M. .; Chen, Y. .; Huang, W. .; Tang, B. .; An, B. .; Han, X. .; Kippax-Jones, M. .; Frogley, M. D. ., et al. Enhanced Benzene Adsorption in Chloro-Functionalized Metal–Organic Frameworks. *J. Am. Chem. Soc.* **2024**, *146*, 28080–28087.

(12) He, T.; Kong, X.-J.; Bian, Z.-X.; Zhang, Y.-Z.; Si, G.-R.; Xie, L.-H.; Wu, X.-Q.; Huang, H.; Chang, Z.; Bu, X.-H., et al. Trace Removal of Benzene Vapour Using Double-Walled Metal–Dipyrazolate Frameworks. *Nat. Mater.* **2022**, *21*, 689–695.

(13) Jajko, G.; Sevillano, J. J. G.; Calero, S.; Makowski, W.; Kozyra, P. The Boost of Toluene Capture in UiO-66 Triggered by Structural Defects or Air Humidity. *J. Phys. Chem. Lett.* **2023**, *14*, 5618–5623.

(14) Gulcay-Ozcan, E.; Iacomi, P.; Rioland, G.; Maurin, G.; Devautour-Vinot, S. Airborne Toluene Detection Using Metal–Organic Frameworks. *ACS Appl. Mater. Interfaces* **2022**, *14*, 53777–53787.

(15) Zheng, X.; Liu, S. .; Rehman, S. .; Li, Z. .; Zhang, P. Highly Improved Adsorption Performance of Metal-Organic Frameworks CAU-1 for Trace Toluene in Humid Air via Sequential Internal and External Surface Modification. *Chem. Eng. J.* **2020**, *389*, 123424.

(16) Pei, J.; Zhang, J. S. Determination of Adsorption Isotherm and Diffusion Coefficient of Toluene on Activated Carbon at Low Concentrations. *Build. Environ.* **2012**, *48*, 66–76.

(17) Berg, F.; Pasel, C. .; Eckardt, T. .; Bathen, D. Temperature Swing Adsorption in Natural Gas Processing: A Concise Overview. *ChemBioEng Rev.* **2019**, *6*, 59–71.

(18) Deng, H.; Pan, T. .; Zhang, Y.; Wang, L.; Wu, Q.; Ma, J.; Shan, W. .; He, H. Adsorptive Removal of Toluene and Dichloromethane from Humid Exhaust on MFI, BEA and FAU Zeolites: An Experimental and Theoretical Study. *Chem. Eng. J.* **2020**, *394*, 124986.

(19) Yu, B.; Deng, H.; Lu, Y.; Pan, T. .; Shan, W. .; He, H. Adsorptive Interaction Between Typical VOCs and Various Topological Zeolites: Mixture Effect and Mechanism. *J. Environ. Sci.* **2024**, *136*, 626–636.

(20) Sui, H.; Liu, H.; An, P.; He, L.; Li, X.; Cong, S. Application of Silica Gel in Removing High Concentrations Toluene Vapor by Adsorption and Desorption Process. *J. Taiwan Inst. Chem. Eng.* **2017**, *74*, 218–224.

(21) Wu, X.-Q.; Huang, D.-D. .; Zhou, Z.-H. .; Dong, W.-W. .; Wu, Y.-P. .; Zhao, J. .; Li, D.-S. .; Zhang, Q. .; Bu, X. Ag-NPs Embedded in Two Novel Zn 3/Zn 5-Cluster-Based Metal–Organic Frameworks for Catalytic Reduction of 2/3/4-Nitrophenol. *Dalton Trans.* **2017**, *46*, 2430–2438.

(22) Alvarez, E.; Guillou, N.; Martineau, C.; Bueken, B.; Van de Voorde, B.; Le Guillouzer, C. .; Fabry, P. .; Nouar, F.; Taulelle, F.; De Vos, D. ., et al. The Structure of the Aluminum Fumarate Metal–Organic Framework A520. *Angew. Chem., Int. Ed.* **2015**, *54*, 3664–3668.

(23) Ramaswamy, P.; Mandal, S. .; Natarajan, S. New Open-Framework Phosphate and Phosphite Compounds of Gallium. *Inorg. Chim. Acta* **2011**, *372*, 136–144.

(24) Volkringer, C.; Loiseau, T. .; Guillou, N. .; Férey, G. .; Haouas, M. .; Taulelle, F. .; Elkaim, E. .; Stock, N. High-Throughput Aided Synthesis of the Porous Metal-Organic Framework-Type Aluminum Pyromellitate, MIL-121, with Extra Carboxylic Acid Functionalization. *Inorg. Chem.* **2010**, *49*, 9852–9862.

(25) Lin, Z.-z.; Jiang, F.-l. .; Yuan, D.-q.; Chen, L. .; Zhou, Y.-f. .; Hong, M.-c. The 3D Channel Framework Based on Indium (iii)–btec, and Its Ion-Exchange Properties (btec= 1, 2, 4, 5-Benzenetetracarboxylate), 2005.

(26) Abrahams, B. F.; Hudson, T. A. .; Robson, R. A New Approach to DCNQI-Based Coordination Polymers via DCNQIH_2. *Cryst. Growth Des.* **2010**, *10*, 1468–1470.

(27) Yang, S.; Ramirez-Cuesta, A. J. .; Newby, R. .; Garcia-Sakai, V. .; Manuel, P. .; Callear, S. K. .; Campbell, S. I. .; Tang, C. C. .; Schröder, M. Supramolecular Binding and Separation of Hydrocarbons Within a Functionalized Porous Metal–Organic Framework. *Nat. Chem.* **2015**, *7*, 121–129.

(28) Wu, J.-Y.; Ding, M.-T. .; Wen, Y.-S. .; Liu, Y.-H. .; Lu, K.-L. Alkali Metal Cation (K+, Cs+) Induced Dissolution/Reorganization of Porous Metal Carboxylate Coordination Networks in Water. *Chem. – Eur. J.* **2009**, *15*, 3604–3614.

(29) Fu, R.; Hu, S. .; Wu, X. Syntheses, Crystal Structures, Thermal Stabilities and Luminescence of Six M(II)-Hydroxyphosphonoacetate Materials. *J. Solid State Chem.* **2011**, *184*, 945–952.

(30) Vougo-Zanda, M.; Huang, J. .; Anokhina, E. .; Wang, X. .; Jacobson, A. J. Tossing and Turning: Guests in the Flexible Frameworks of Metal (III) Dicarboxylates. *Inorg. Chem.* **2008**, *47*, 11535–11542.

(31) Li, S.; Subhan, S.; Zhou, L.; Li, J.; Zhao, Z.; Zhao, Z. High Efficiency of Toluene Ad-/Desorption on Thermal-Conductive HKUST-1@ BN Nanosheets Composite. *Chem. Eng. J.* **2023**, *465*, 142791.

(32) Hu, L.; Wu, W. .; Gong, L.; Zhu, H.; Jiang, L.; Hu, M. .; Lin, D.; Yang, K. A Novel Aluminum-Based Metal-Organic Framework with Uniform Micropores for Trace BTEX Adsorption. *Angew. Chem.* **2023**, *135*, e202215296.

(33) Xie, L.-H.; Liu, X.-M.; He, T.; Li, J.-R. Metal-Organic Frameworks for the Capture of Trace Aromatic Volatile Organic Compounds. *Chem* **2018**, *4*, 1911–1927.

(34) Lillo-Ródenas, M.; Cazorla-Amorós, D.; Linares-Solano, A. Benzene and Toluene Adsorption at Low Concentration on Activated Carbon Fibres. *Adsorption* **2011**, *17*, 473–481.

(35) Li, S. Preparation and Characterization of Bimetal MOF-74-Co/Cu and Its Toluene Adsorption Performances. *J. Porous Mater.* **2023**, *30*, 421–432.

(36) Ma, X.; Wang, W.; Sun, C.; Li, H.; Sun, J.; Liu, X. Adsorption Performance and Kinetic Study of Hierarchical Porous Fe-Based MOFs for Toluene Removal. *Sci. Total Environ.* **2021**, *793*, 148622.

(37) Charalambous, C.; Moubarak, E.; Schilling, J.; Sanchez Fernandez, E.; Wang, J.-Y.; Herraiz, L. .; Mcilwaine, F.; Peh, S. B.; Garvin, M.; Jablonka, K. M., et al. A Holistic Platform for Accelerating Sorbent-Based Carbon Capture. *Nature* **2024**, *632*, 89–94.

(38) Perdew, J. P.; Burke, K.; Ernzerhof, M. Generalized Gradient Approximation Made Simple. *Phys. Rev. Lett.* **1996**, *77*, 3865.

(39) Grimme, S.; Ehrlich, S.; Goerigk, L. Effect of the Damping Function in Dispersion-Corrected Density Functional Theory. *J. Comput. Chem.* **2011**, *32*, 1456–1465.

(40) Dubbeldam, D.; Calero, S.; Ellis, D. E.; Snurr, R. Q. RASPA: Molecular Simulation Software for Adsorption and Diffusion in Flexible Nanoporous Materials. *Mol. Simul.* **2016**, *42*, 81–101.

(41) LSMO, T.; team, A. An AiiDA Workflows for the LSMO Laboratory at EPFL, Accessed: 2023, 2023.

(42) Huber, S. P.; Zoupanos, S. .; Uhrin, M. .; Talirz, L. .; Kahle, L. .; Häuselmann, R. .; Gresch, D. .; Müller, T. .; Yakutovich, A. V. .; Andersen, C. W. ., et al. AiiDA 1.0, a Scalable Computational Infrastructure for Automated Reproducible Workflows and Data Provenance. *Sci. Data* **2020**, *7*, 300.

(43) Uhrin, M.; Huber, S. P. .; Yu, J. .; Marzari, N. .; Pizzi, G. Workflows in AiiDA: Engineering a High-Throughput, Event-Based Engine for Robust and Modular Computational Workflows. *Comput. Mater. Sci.* **2021**, *187*, 110086.

(44) Manz, T. A.; Sholl, D. S. Chemically Meaningful Atomic Charges That Reproduce the Electrostatic Potential in Periodic and Nonperiodic Materials. *J. Chem. Theory Comput.* **2010**, *6*, 2455–2468.

(45) Maerzke, K. A.; Schultz, N. E. .; Ross, R. B. .; Siepmann, J. I. TraPPE-UA Force Field for Acrylates and Monte Carlo Simulations for Their Mixtures with Alkanes and Alcohols. *J. Phys. Chem. B* **2009**, *113*, 6415–6425.

(46) Wick, C. D.; Siepmann, J. I. .; Klotz, W. L. .; Schure, M. R. Temperature Effects on the Retention of n-Alkanes and Arenes in Helium–Squalane Gas–Liquid Chromatography: Experiment and Molecular Simulation. *J. Chromatogr. A* **2002**, *954*, 181–190.

(47) Rappe, A. K.; Casewit, C. J.; Colwell, K. S.; Goddard, W. A.; Skiff, W. M. UFF, a Full Periodic Table Force Field for Molecular Mechanics and Molecular Dynamics Simulations. *J. Am. Chem. Soc.* **1992**, *114*, 10024–10035.

(48) Li, Y.; Jin, X. .; Moubarak, E. .; Smit, B. A Refined Set of Universal Force Field Parameters for Some Metal Nodes in Metal–Organic Frameworks. *J. Chem. Theory Comput.* **2024**, *20*, 10540–10552.

(49) Willems, T. F.; Rycroft, C. H.; Kazi, M. .; Meza, J. C. .; Haranczyk, M. Algorithms and Tools for High-Throughput Geometry-Based Analysis of Crystalline Porous Materials. *Microporous Mesorous Mater.* **2012**, *149*, 134–141.

(50) Sharpe, S. W.; Sams, R. L. .; Johnson, T. J. In *Applied Imagery Pattern Recognition Workshop, 2002. Proceedings.* 2002, pp 45–48.

(51) Harrick, N.; Du Pre, F. Effective Thickness of Bulk Materials and of Thin Films for Internal Reflection Spectroscopy. *Appl. Opt.* **1966**, *5*, 1739–1743.
